# Crystal Structures of R-Type Bacteriocin Sheath and Tube Proteins CD1363 and CD1364 From *Clostridium difficile* in the Pre-assembled State

**DOI:** 10.3389/fmicb.2018.01750

**Published:** 2018-08-03

**Authors:** Nina Schwemmlein, Jan Pippel, Emerich-Mihai Gazdag, Wulf Blankenfeldt

**Affiliations:** ^1^Structure and Function of Proteins, Helmholtz Centre for Infection Research, Braunschweig, Germany; ^2^Institute for Biochemistry, Biotechnology and Bioinformatics, Technische Universität Braunschweig, Braunschweig, Germany

**Keywords:** diffocin, *Clostridium difficile*, phage tail-like bacteriocins, R-type, crystal structure, sheath, tube

## Abstract

Diffocins are high-molecular-weight phage tail-like bacteriocins (PTLBs) that some *Clostridium difficile* strains produce in response to SOS induction. Similar to the related R-type pyocins from *Pseudomonas aeruginosa*, R-type diffocins act as molecular puncture devices that specifically penetrate the cell envelope of other *C. difficile* strains to dissipate the membrane potential and kill the attacked bacterium. Thus, R-type diffocins constitute potential therapeutic agents to counter *C. difficile*-associated infections. PTLBs consist of rigid and contractile protein complexes. They are composed of a baseplate, receptor-binding tail fibers and an inner needle-like tube surrounded by a contractile sheath. In the mature particle, the sheath and tube structure form a complex network comprising up to 200 copies of a sheath and a tube protein each. Here, we report the crystal structures together with small angle X-ray scattering data of the sheath and tube proteins CD1363 (39 kDa) and CD1364 (16 kDa) from *C. difficile* strain CD630 in a monomeric pre-assembly form at 1.9 and 1.5 Å resolution, respectively. The tube protein CD1364 displays a compact fold and shares highest structural similarity with a tube protein from *Bacillus subtilis* but is remarkably different from that of the R-type pyocin from *P. aeruginosa*. The structure of the R-type diffocin sheath protein, on the other hand, is highly conserved. It contains two domains, whereas related members such as bacteriophage tail sheath proteins comprise up to four, indicating that R-type PTLBs may represent the minimal protein required for formation of a complete sheath structure. Comparison of CD1363 and CD1364 with structures of PTLBs and related assemblies suggests that several conformational changes are required to form complete assemblies. In the sheath, rearrangement of the flexible N- and C-terminus enables extensive interactions between the other subunits, whereas for the tube, such contacts are primarily established by mobile α-helices. Together, our results combined with information from structures of homologous assemblies allow constructing a preliminary model of the sheath and tube assembly from R-type diffocin.

## Introduction

In order to infect bacteria, bacteriophages use an attachment organelle known as the “tail,” which recognizes the host cell and attaches the phage’s capsid to it. The tail then acts as a molecular syringe to puncture the cell envelope and establish an inner channel that translocates genomic DNA and proteins from the capsid into the cell cytoplasm (Goldberg et al., 1994; Leiman and Shneider, 2012; Hu et al., 2015). Tailed bacteriophages (order *Caudovirales*) can be divided into three families: *Myoviridae* (long contractile tail), *Siphoviridae* (long non-contractile tail), and *Podoviridae* (short non-contractile tail) (Sharp, 2001; Ackermann, 2003; Veesler and Cambillau, 2011).

Interestingly, bacterial genomes often contain gene clusters encoding for structural elements that are evolutionary related to bacteriophage tail structures with regard to morphology, size, and the mechanism of action but without containing a phage capsid. This includes, e.g., the T6SS (Leiman et al., 2009), needle-like particles such as the Afp from *Serratia entomophila* (Hurst et al., 2004) or the PVC (Yang et al., 2006), as well as PTLBs such as the “pyocins” from *Pseudomonas aeruginosa* or the “monocins” from *Listeria monocytogenes* (Nakayama et al., 2000; Lee et al., 2016). PTLBs, also referred to as tailocins, are high-molecular weight protein particles that are widespread in bacteria and for a subset of which antibacterial activity against competing strains of the same species has been demonstrated (Ghequire and Mot, 2015; Scholl, 2017). Two types of PTLBs have been identified: the R-type PTLBs, which are rigid and contractile and are related to *Myoviridae* tail structures (Kageyama, 1964; Ishii et al., 1965), and the F-type PTLBs, which are flexible and non-contractile and are related to *Siphoviridae* tails (Takeya et al., 1967; Govan, 1974; Kuroda and Kageyama, 1979). Both types are exemplified by the so far best-studied PTLBs, namely the R- and F-type pyocins from *P. aeruginosa* (Nakayama et al., 2000; Michel-Briand and Baysse, 2002; Scholl, 2017). A rather recently identified group of PTLBs are the R-type “diffocins” from *Clostridium difficile*, which constitute the only Gram-positive R-type PTLBs known to date (Gebhart et al., 2012; Scholl, 2017).

R-type PTLBs share a common structure and mechanism with bacteriophage tails of the *Myoviridae* family and although they cannot independently replicate, it has been suggested that PTLBs should not be considered defective prophages but rather as specifically adapted to their function as antibacterial instruments (Nakayama et al., 2000; Leiman et al., 2009; Ghequire and Mot, 2015). R-type PTLBs are produced and accumulate within the bacterium under SOS response conditions and are only released upon lysis of the cell (Matsui et al., 1993; Williams et al., 2008; Lee et al., 2016). Whereas for members of other strains from the same species, contact with only a single particle can lead to cell death (Kageyama et al., 1964), cells harboring the respective PTLB genes themselves are provided with a resistance mechanism (Scholl et al., 2009; Köhler et al., 2010). Thus, sister cells of the same strain gain advantage from self-sacrifice of one member during which PTLBs are released (Lee et al., 2016; Scholl, 2017).

**FIGURE 1:**
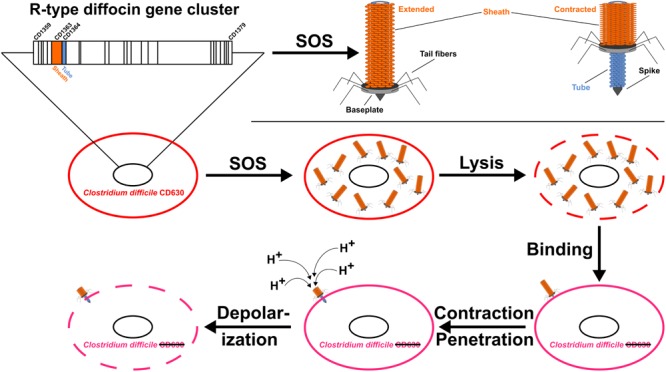
The genome of *Clostridium difficile* strain CD630 contains a 25-gene cluster encoding the R-type “diffocin.” Diffocins form after SOS induction and are released by cell lysis to bind closely related but unprotected *C. difficile* strains. Contraction of the diffocin sheath (orange) drives the tube (blue) through the wall of the attacked cell and leads to death by dissipation of the membrane potential.

All R-type PTLBs consist of an inner needle-like rigid tube surrounded by a contractile sheath. Both of these complexes are composed of disks that assemble from six sheath or tube subunits via an axial sixfold rotational symmetry and are stacked atop each other after right-handed rotation (Heymann et al., 2013; Ge et al., 2015; Nováček et al., 2016; Brackmann et al., 2017; Salih et al., 2018). With up to 200 copies of the individual subunits in one sheath or tube assembly (Takeda and Kageyama, 1975), these proteins constitute the main body of the particle, which is capped by a complex baseplate structure with six attached receptor-binding proteins (Ge et al., 2015; Taylor et al., 2018). These proteins are responsible for recognition and binding to specific receptors on the surface of the target cell and thus determine the very narrow specificity spectrum for a subset of strains within the species (Meadow and Wells, 1978; Nakayama et al., 2000; Williams et al., 2008; Köhler et al., 2010; Gebhart et al., 2012; Kocíncová and Lam, 2013). Binding triggers extensive conformational changes in the baseplate, accompanied by contraction of the sheath, which, due to extensive interactions, drives the rigid inner tube into the cell envelope. The driving force of sheath contraction and subsequent movement of the tube resides in the high-energy metastable structure of the extended state of the sheath (Aksyuk et al., 2009; Leiman and Shneider, 2012; Ge et al., 2015; Taylor et al., 2016; Brackmann et al., 2017). As a consequence of the puncturing, ions can pass through the tube, resulting in fast dissipation of the membrane potential and death of the attacked cell (**Figure 1**) (Uratani and Hoshino, 1984; Strauch et al., 2001). Thus, the complex network between the contractile sheath and the rigid tube is essential for the killing potency of R-type PTLBs (Ge et al., 2015; Kube and Wendler, 2015; Brackmann et al., 2017; Scholl, 2017).

Their impressive killing capacity makes R-type PTLBs promising candidates for an efficient treatment of bacterial infections and, given their narrow strain-specificity, it seems likely that they can be administered without the risk of transmissible resistance or adverse effects on the beneficial flora (Ghequire and De Mot, 2014; Behrens et al., 2017; Scholl, 2017). Additionally, several studies demonstrated that R-type PTLBs can be specifically bioengineered to target also other species or strains (Williams et al., 2008; Scholl et al., 2009, 2012), and prove-of-concept studies have been conducted to treat the respective infections (Merrikin and Terry, 1972; Haas et al., 1974; Scholl and Martin, 2008; Ritchie et al., 2011; Gebhart et al., 2015). Thus, R-type PTLBs could serve as novel therapeutics to treat infections particularly caused by antibiotic-resistant bacteria.

The anaerobic Gram-positive bacterium *C. difficile* is a human pathogen that can be found in the intestine of humans as well as animals (Hall and O’toole, 1935; Larson et al., 1977; George et al., 1978; Bartlett, 1994). Symptoms of infections range from diarrhea to life-threatening pseudomembranous colitis (Poutanen and Simor, 2004; Borali and Giacomo, 2016) and *C. difficile*-associated morbidity and mortality in humans has increased in recent years (Lessa et al., 2015). *C. difficile* and its spores are resistant to a range of antibiotics as well as to various environmental factors and can therefore form reservoirs in healthcare settings, which significantly enhances the risk for hospital-acquired infections (Bignardi, 1998; Zilberberg et al., 2008; Vohra and Poxton, 2011; Tenover et al., 2012; Slimings and Riley, 2014). Additionally, a preceding treatment with broad-spectrum antibiotics constitutes a major risk factor for CDIs since the protective microbiome in the gut is disrupted, providing a less competitive habitat for relapsing *C. difficile* (Theriot and Young, 2015). Therefore, alternatives to conventional antibiotics are urgently needed to specifically target *C. difficile*, and the R-type diffocins, possibly after bioengineering, constitute promising candidates (Gebhart et al., 2015). Toward this, insights into their three-dimensional structures could provide valuable information about their activity, assembly, and specificity. In this respect, R-type pyocins from *P. aeruginosa* are currently the most thoroughly studied R-type PTLBs, whereas structural information for other particles such as the *C. difficile* R-type diffocins is largely absent. Additionally, in contrast to prophage-like elements and bacteriophage tails, no crystal structures of the free sheath and tube building blocks are available, which precludes insights into the pre-assembly states of these proteins. Here, we therefore aimed at determining the crystal structures of these components from *C. difficile* strain CD630 individually. By extensive comparison with homologous structures, we demonstrate that several conformational changes are required in order to form the R-type diffocin particle. We also provide a model of the assembled sheath and tube structures of the R-type diffocin contractile apparatus, which may serve as templates for future cryo electron microscopy (cryo-EM) studies.

## Materials and Methods

### Cloning, Expression, and Purification

The genes encoding the R-type diffocin sheath protein CD1363 and tube protein CD1364 were PCR-amplified from genomic *C. difficile* CD630 DNA, kindly provided by Prof. Dr. Ralf Gerhard (Hannover Medical School, Hannover, Germany). Both genes were subcloned into a pOPINM expression vector (Addgene plasmid # 26044; a kind gift from Ray Owens; Berrow et al., 2007) via SLIC (Li and Elledge, 2007), using appropriate oligonucleotide primers listed in Supplementary Table S1 to yield fusion proteins with N-terminal 6xHis-MBP-tags and HRV3C protease cleavage sites. Positive clones of both constructs were identified with colony PCR and the correct sequence was verified via sequencing. Optimal conditions for recombinant protein production were determined in small-scale test expression experiments and large-scale expression was performed with *Escherichia coli* BL21-CodonPlus-RIL cells (Agilent Technologies, Santa Clara, CA, United States) for 24 h in M9 minimal medium (Miller, 1972) at 20°C for seleno-L-methionine-(SeMet-)labeled sheath protein CD1363 or in auto-induction medium (Studier, 2014) at 25°C for native tube protein CD1364. Cell pellets were resuspended in 20 mM HEPES, 300 mM NaCl, 3 mM DTT, pH 7.5, homogenized and centrifuged. The supernatants were applied onto a 5 mL MBPTrap HP column on an ÄKTA system (GE Healthcare Life Sciences, Pittsburgh, PA, United States) and eluted with 10 mM maltose in the same buffer. Tags were cleaved off with HRV3C protease at 4°C and overnight incubation. To subsequently remove cleaved affinity tags and uncleaved protein, a nickel affinity chromatography step was performed via a 5 mL HisTrap^TM^ HP chelating column (GE Healthcare Life Sciences) and the flow-through containing the respective protein was concentrated using a Vivaspin 6 10 kDa cutoff concentrator (GE Healthcare Life Sciences). As a polishing step, the proteins were passed through a Superdex 75 16/60 or 26/60 prep grade column (GE Healthcare Life Sciences) and fractions containing the proteins of interest were concentrated and dialyzed against 20 mM HEPES, 300 mM NaCl, 3 mM DTT, pH 7.5. Protein purity was assessed by SDS-PAGE and protein concentrations were determined spectroscopically using calculated extinction coefficients based on the amino acid sequence via the PROTPARAM web server^1^ (Gasteiger et al., 2005). The correct molecular masses of the purified proteins were confirmed at the Department Chemical Biology at the Helmholtz Center for Infection Research, using a maXis HD ultra-high resolution (UHR) Q-TOF mass spectrometer equipped with a Apollo II electrospray source (Bruker Daltoniks, Bremen, Germany) and an Ultimate 3000RS autosampler together with a binary high gradient pump (Dionex/Thermo Fisher Scientific, Waltham, MA, United States). A solvent flow of 50 μL/min was used for infusion of a calibrating solution as well as for the sample. Data were processed using the Data Analysis Software Version 4.2 (Bruker Daltoniks). Original mass spectra containing peaks from multiply charged ions were smoothed and deconvoluted to obtain a singly charged mass spectrum. The UV traces of the size exclusion polishing step, SDS PAGE analysis of purified proteins and ESI-MS spectra are shown in Supplementary Figure S1.

### Small Angle X-Ray Scattering (SAXS) Analysis

The oligomeric states of the purified proteins were assessed by SAXS experiments at beamline BM29 at the European Synchrotron Radiation Facility (ESRF, Grenoble, France; Pernot et al., 2013) equipped with a PILATUS 1M detector and by using a sample detector distance of 2.867 m and a wavelength of λ = 0.9919. All data collection steps were carried out at 4°C and scattering data from buffer ingredients were subtracted from protein scattering data using PRIMUS software (Konarev et al., 2003). Best data were obtained in 20 mM HEPES pH 7.5, 100 mM NaCl, 3 mM DTT and with 0.63 and 1.25 mg/mL of sheath protein CD1363 or tube protein CD1364, respectively. Scattering data were normalized, averaged and merged using PRIMUS software and SAXS envelopes were calculated with DAMMIF, DAMSEL, DAMSUP, DAMAVER, and DAMFILT of the ATSAS software package (Franke and Svergun, 2009). Fitting of the respective crystal structures with the experimental scattering curves and with the calculated envelopes was performed with CRYSOL (Svergun et al., 1995) or with UCSF Chimera (Pettersen et al., 2004), respectively.

### Protein X-Ray Crystallography and Structure Determination

Vapor diffusion experiments were set up in a 96-well sitting drop format at 19°C using a dispensing robot (Zinsser Analytics, Frankfurt, Germany). 0.1 μL protein solution at a concentration of 20 mg/mL for both the SeMet-labeled sheath protein CD1363 and native tube protein CD1364 were mixed with an equal amount of reservoir to give concentrations listed in **Table 1**. The drops were equilibrated against 70 μL of reservoir solution and crystals appeared after a few days. Before flash cooling the crystals in liquid nitrogen, 10% (v/v) of (*2R,3R*)-(-)-2,3-butanediol was added as cryoprotectant.

**Table 1:** Crystallization, data collection and refinement statistics, ^1^values in parenthesis correspond to highest resolution shell.

	SeMet-labeled CD1363 (PDB: 6GKW)	Native CD1364 (PDB: 6GKX)
**Crystallization and data collection**		
Crystallization buffer	0.1 M Bis-Tris pH 5.5, 25% (w/v) PEG 3350	20% (w/v) PEG 3350, 0.2 M ammonium chloride
Crystallization drop	0.1 μL crystallization buffer + 0.1 μL of 20 mg/mL protein in 20 mM HEPES, 300 mM NaCl, 3 mM DTT, pH 7.5	0.1 μL crystallization buffer + 0.1 μL of 20 mg/mL protein in 20 mM HEPES, 300 mM NaCl, 3 mM DTT, pH 7.5
Wavelength (Å)/beamline	0.97945/SLS PXII	0.918/BESSY II 14.1
Resolution range (Å)	40.49–1.90 (1.94–1.90)	43.36–1.50 (1.53–1.50)
Space group	C121	P4_3_2_1_2
Unit cell parameters a, b, c (Å) α, β, γ (°)	75.9, 47.9, 112.5 90.0, 92.3, 90.0	36.3, 36.3, 216.8 90.0, 90.0, 90.0
Mosaicity (°)	0.22	0.10
Total no. of measured reflections	432621 (27802)	613640 (30875)
Unique reflections	32088 (2087)	24715 (1195)
Multiplicity	13.5 (13.3)	24.8 (25.9)
Mean I/σ(I)	21.5 (1.8)	19.7 (1.7)
Completeness (%)	99.9 (99.8)	100.0 (100.0)
R*_meas_* (%)	8.8 (156.6)	8.5 (233.5)
R*_p.i.m._* (%)	2.4 (42.1)	1.7 (45.5)
CC1/2 (%)	100.0 (72.6)	100.0 (70.4)
Wilson B-factor (Å^2^)	32.20	20.97
Monomers/asymmetric unit	1	1
**Refinement**		
Resolution range (Å)	38.36–1.90 (1.96–1.90)	35.83–1.50 (1.57–1.50)
R_work_ (%)	21.31 (31.66)	19.15 (26.78)
R_free_ (%)	24.85 (36.22)	22.00 (30.95)
No. of non-H atoms		
Protein	2705	1075
Water	180	129
RMS deviation		
Bonds (Å)	0.003	0.010
Angles (°)	0.518	1.043
Average B factors (Å^2^)		
Protein	51.58	37.07
Water	45.56	38.29
All atoms	51.21	37.20
Ramachandran plot		
Favored regions (%)	97.4	97.7
Allowed regions (%)	2.6	2.3
Outliers (%)	0.0	0.0

X-ray data collection for native tube protein CD1364 was carried out at 100 K at beamline 14.1 at BESSY II (Helmholtz-Zentrum Berlin (HZB), Berlin, Germany; Gerlach et al., 2016). For SeMet-labeled sheath protein CD1363, a SAD data set was collected on beamline PXII at the Swiss Light Source (SLS; Paul Scherrer Institut, Villigen, Switzerland; Fuchs et al., 2014). Diffraction data were indexed, integrated and scaled with XDS (Kabsch, 2010) and AIMLESS (Evans and Murshudov, 2013) from the CCP4 suite (Winn et al., 2011). Details of data collection are listed in **Table 1**. The structure of SeMet-labeled sheath protein CD1363 was determined using AUTOSOL (Terwilliger et al., 2009). Eight SeMet sites were identified, indicating the presence of one molecule in the asymmetric unit (Matthews, 1968; Winn et al., 2011). The structure of the native tube protein CD1364 was solved by molecular replacement with PHASER (McCoy et al., 2007), using a truncated model of a monomer of P54332 from *Bacillus subtilis* (*B. subtilis*; PDB: 2GUJ; unpublished). Initial model building was performed using AUTOBUILD (Terwilliger et al., 2008) and final models were obtained by iterative cycles of TLS-motion refinement with PHENIX.REFINE (Afonine et al., 2012) and manual rebuilding using COOT (Emsley et al., 2010). All programs were run through the PHENIX suite (Adams et al., 2010). Coordinates of the SeMet-labeled sheath protein CD1363 and tube protein CD1364 structures are available at the PDB^2^ (Berman et al., 2000) under accession codes 6GKW and 6GKX and refinement statistics are summarized in **Table 1**. Secondary structure elements were assigned using the DSSP web server (Kabsch and Sander, 1983; Touw et al., 2015). The DALI web server ^3^ was used to analyze structural similarities and to calculate Z-scores as well as sequence similarities toward homologous proteins (Holm and Laakso, 2016). Sequence alignments were generated with the PROMALS3D web server ^4^ (Pei et al., 2008) and alignment figures were prepared with the ESPript 3.0 web server ^5^ (Robert and Gouet, 2014) after manual editing. Structural figures were prepared using PyMOL (The PyMOL Molecular Graphics System Version 1.8.2.3; Schrödinger LLC, 2010) including the APBS plugin for electrostatic potential surface presentations (Baker et al., 2001). Topology diagrams were generated using TopDraw (Bond, 2003).

### Structure Modeling of the Sheath and Tube Assemblies in Extended R-Type Diffocin

Structural alignments were generated by superimposing the sheath and tube proteins CD1363 and CD1364 on monomers of the R-type pyocin sheath protein FIR2 (PDB: 3J9Q; Ge et al., 2015) or the bacteriophage φ812 tail tube protein gp104 (PDB: 5LI2; Nováček et al., 2016) via the DALI web server (Holm and Laakso, 2016). Using PyMOL (The PyMOL Molecular Graphics System Version 1.8.2.3; Schrödinger LLC, 2010) as well as the protein comparison tool from the RCSB PDB^6^, the obtained alignments were visually inspected and manually adjusted. Models of tube and sheath assembles were generated on the basis of the R-type pyocin sheath (PDB: 3J9Q; Ge et al., 2015) and the bacteriophage φ812 tail tube (PDB: 5LI2; Nováček et al., 2016) using MODELLER (Webb and Šali, 2016). The models were further treated by two cycles of VTFM-based optimization and MD-based refinement in slow mode (Šali and Blundell, 1993) as implemented in MODELLER (Webb and Šali, 2016). Loops and secondary structure elements that exhibited severe clashes such as residues F36–G55, Y65–S68, and V121–G125 of the tube protein CD1364 and residues Y308–E326 of the sheath protein CD1363 were excluded from the assembly. Since the 5LI2 template used for modeling of the tube disk comprises a single layer only, generation of two additional disks was achieved via superimposition of both single disks of R-type diffocin sheath and tube onto 5LI2 and their subsequent merging. Afterward, the merged model contained both an R-type diffocin sheath and tube and was superimposed back onto two adjacent R-type diffocin disks of the pyocin-based sheath model such that the three-layered tube structure follows the sheath geometry of this model. Figures were generated as described above.

## Results and Discussion

### Cloning, Expression, and Purification

Genes of CD1363 and CD1364 from *C. difficile* strain 630 were successfully PCR-amplified from genomic DNA and subcloned into a pOPINM expression vector (Berrow et al., 2007) via SLIC (Li and Elledge, 2007), using the oligonucleotide primers listed in Supplementary Table S1. Positive clones for both constructs were identified via colony PCR and the correct sequences were confirmed via sequencing.

During test expression experiments, the highest yields of soluble protein were obtained with *E. coli* BL21-CodonPlus-RIL cells in auto-induction medium (Studier, 2014) for both proteins. However, since SeMet-labeling was required to determine the structure of sheath protein CD1363, large-scale expression for this protein was performed in M9 minimal medium (Miller, 1972).

The obtained fusion proteins comprised a combination of an N-terminal 6xHis and an MBP-tag followed by a HRV3C protease cleavage site to remove both tags simultaneously. The proteins were affinity-purified using an MBPTrap HP column (GE Healthcare Life Sciences, Pittsburgh, PA, United States). Subsequently, the N-terminal 6xHis-MBP-tags were cleaved off with HRV3C protease during overnight incubation and uncleaved fusion protein along with free N-terminal tags were removed using a 5 mL HisTrap^TM^ HP column (GE Healthcare Life Sciences). Tag-free CD1363 (˜39 kDa) and CD1364 (˜16 kDa) were identified in the flow-through, concentrated and then finally purified via gel filtration, in which both proteins eluted as single prominent peaks (Supplementary Figure S1A). Via mass spectrometry analysis using electrospray ionization (ESI)-TOF, the full integrity and correct molecular weight were confirmed for the pooled fractions (CD1363: theoretical mass: 39344.5547 Da, experimental mass: 39345.3228 Da; CD1364: theoretical mass: 16161.4824 Da, experimental mass: 16161.8672 Da; Supplementary Figure S1B).

### General Organization of the R-Type Diffocin Tube Protein CD1364

The R-type diffocin tube protein CD1364 crystallized in space group P4_3_2_1_2 containing one molecule in the asymmetric unit. Initial phases were obtained by molecular replacement using a truncated model of a single central β-barrel from the phage-like element P54332 protein XkdM from *B. subtilis* (PDB: 2GUJ, unpublished), and the structural model was subsequently refined to a resolution of 1.5 Å with R_work_ = 19.15% and R_free_ = 22.00% (**Table 1**).

**FIGURE 2:**
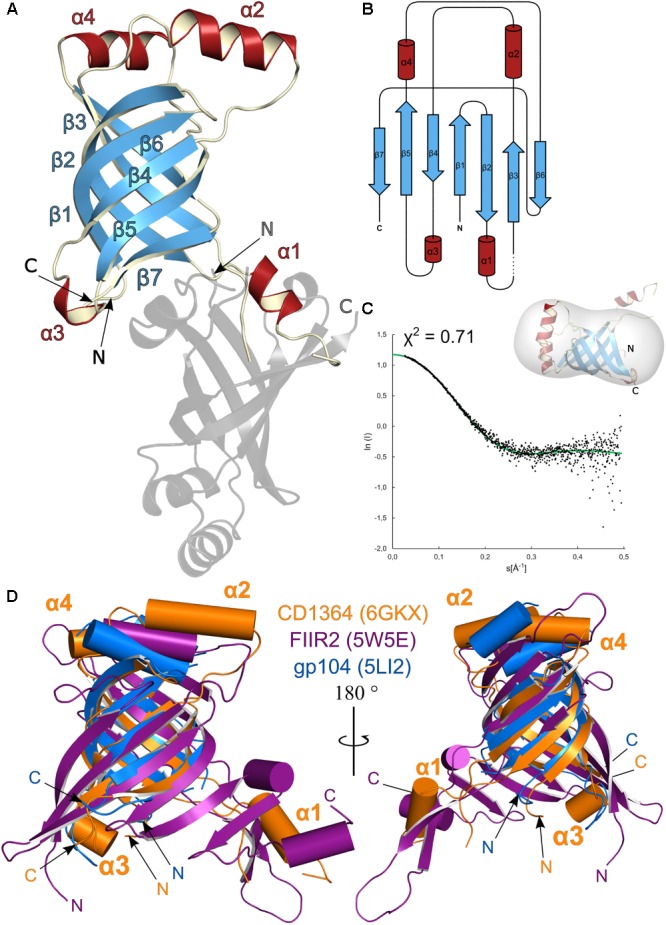
Structural analysis of *Clostridium difficile* R-type diffocin tube protein CD1364. The structures of monomeric CD1364 (blue and red) and a symmetry-related molecule (gray) are shown as cartoon representation in **(A)** and the corresponding topology diagram is shown in **(B)**. Secondary structure motifs are numbered consecutively. SAXS analysis of purified CD1364 is presented in **(C)** with a fit of the experimental SAXS data (black dots) and a theoretical curve calculated from the crystal structure (green dots) together with the CD1364 crystal structure fitted to the calculated SAXS envelope. In **(D)**, monomeric structures from homologous tube assembles of R-type pyocin (FIIR2; PDB: 5W5E; Zheng et al., 2017) and the bacteriophage φ812 tail (gp104; PDB: 5LI2; Nováček et al., 2016) were superimposed onto CD1364. N- and C-termini are labeled with N and C, respectively.

CD1364 possesses a compact fold with a central β-barrel flanked by two α-helices at both of its open sides (**Figures 2A,B**). Whereas α-helices α2 and α4 cap the β-barrel on one side, the α-helices at the other protrude away from the β-barrel such that α1 covers the β-barrel of a symmetry-related neighbor (**Figure 2A**). Higher B-factors indicate increased flexibility for α1, which hints at structural changes that may occur during tube assembly.

As was suspected from the molecular replacement process, CD1364 shares highest structural and sequence similarity with XkdM from *B. subtilis* as well as with gp104 from the bacteriophage φ812 tail tube (Nováček et al., 2016) (Supplementary Table S2), and the overall fold for these proteins is mainly preserved (Supplementary Figure S2). Conserved residues are primarily identified within β1, β2, β3, and β6 as well as in α1 and α2 (Supplementary Figure S3). Notably, in XkdM, α1 does not protrude away from the core of the structure such as in the crystal structure of CD1364 discussed here, but rather caps the β-barrel of the same molecule, again suggesting a high degree of flexibility for this region in both CD1364 and XkdM (Supplementary Figure S2).

Although the corresponding sequence identity is low, tube structures of other bacteriophage tails, bacterial T6SSs and R-type pyocin can be superimposed with reasonable rmsd onto CD1364 (**Figure 2D** and Supplementary Table S2). These structures all have a central β-sheet architecture with at least one α-helix (α2 in CD1364) in common (Brackmann et al., 2017). However, the β-sheet does not appear as compact as in CD1364 and can rather be described as two β-sheets tilted by an angle of 90° (Ge et al., 2015) among the less related homologs such as FIIR2 from R-type pyocin (PDB: 3J9Q, 5W5E; Ge et al., 2015; Zheng et al., 2017), the T6SS hemolysin co-regulated tube protein 1 (HCP1) from *Burkholderia pseudomallei* (PDB: 3WX6; Lim et al., 2015) or phage tail tube protein gp19 from bacteriophage T4 (PDB: 5W5F; Zheng et al., 2017; Supplementary Figure S2). In addition, several differing loop conformations as well as insertions of secondary structure elements are observed. Particularly, a prominent loop (K33–G58 in CD1364) is variable and folds into a flexible α-helix in CD1364 (α1), XkdM from *B. subtilis* and HCP1 from the T6SS of *B. pseudomallei* (Lim et al., 2015), whereas these residues form a protruding β-hairpin in FIIR2 as well as in gp19 from bacteriophage T4 (Zheng et al., 2017; **Figure 2D** and Supplementary Figure S2). Interestingly, the number of amino acids of this loop is nearly unchanged (25–28) and although several residues from this region contribute to inter-disk contacts in the assembled tubes of bacterial T6SSs, the tail of bacteriophage T4 as well as R-type pyocin (Ge et al., 2015; Lim et al., 2015; Wang et al., 2017; Zheng et al., 2017), the corresponding sequence is not conserved (Lim et al., 2015), indicating potentially different principles of tube assembly in these particles.

### General Organization of the R-Type Diffocin Sheath Protein CD1363

The R-type diffocin sheath protein CD1363 crystallized in space group C121 with one molecule in the asymmetric unit. Despite extensive attempts, it was not possible to determine the structure via molecular replacement. Therefore, SAD data were collected from a crystal obtained from SeMet-labeled CD1363 protein. These crystals were of the same space group and exhibited identical cell parameters, allowing for straightforward structure solution and refinement. Because crystals of the labeled protein diffracted to higher resolution, the structure was refined against these data (**Table 1**) and the subsequent discussion refers to SeMet-labeled CD1363.

**FIGURE 3:**
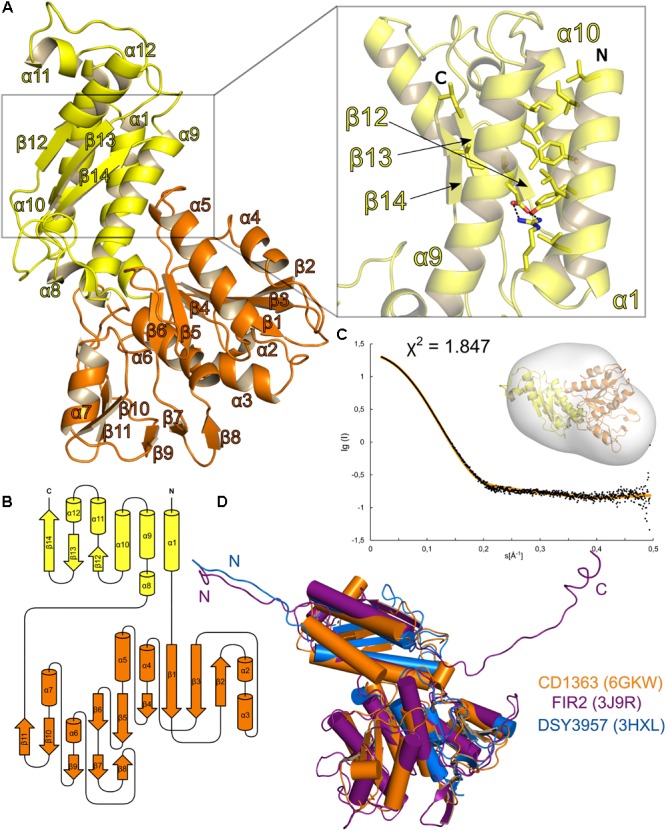
Structural analysis of *Clostridium difficile* R-type diffocin sheath protein CD1363. The structure of monomeric CD1363 is shown as cartoon representation in **(A)** with Domain I and II colored in yellow and orange, respectively. Domain I contains a central three-helix bundle that is stabilized by hydrophobic interactions and by a salt bridge (see insert). The C-terminal part of this domain is organized as a three-stranded β-sheet that hydrophobically interacts with the helix bundle. The general domain organization of CD1363 is shown as topology diagram in **(B)**. SAXS analysis of purified CD1363 is presented in **(C)** with a fit of the experimental SAXS data (black dots) and a theoretical curve calculated from the crystal structure (orange dots) as well as a fit of the CD1364 crystal structure to the SAXS envelope. In **(D)**, monomeric structures of homologous sheath proteins from assembled R-type pyocin (FIIR2; PDB: 3J9R; Ge et al., 2015) and from monomeric DSY3957 from *Desulfitobacterium hafniense* (PDB: 3HXL; Aksyuk et al., 2011) were superimposed onto CD1363. N- and C-termini are labeled with N and C, respectively.

In the crystal form discussed here, CD1363 is monomeric and comprises two domains (**Figure 3A**). The domain arrangement is best described as a “Russian doll” system (Aksyuk et al., 2009, 2011) in which domain II (residues G26–S225; domain numbering according to Leiman and Shneider, 2012) is an insertion into domain I (residues G4–R25 and residues L226–I354; **Figure 3B** and Supplementary Figure S4). At the N-terminus, residues P6–A25 form a long α-helix that closely interacts with α-helices α9 and α10, which flank the central antiparallel three-stranded β-sheet. Domain I is furthermore completed by three smaller α-helices. The larger domain II is composed of a central six-stranded β-sheet and five smaller β-strands flanked by four and two α-helices, respectively (**Figure 3A**).

Despite relatively low sequence identities, several PDB entries related to sheath protein CD1363 were identified via the DALI web server (Holm and Laakso, 2016), and the corresponding rmsd as well as Z-scores indicate a conserved fold for domain I and II of CD1363 (Supplementary Figure S4 and Supplementary Table S3). However, apart from the R-type pyocin sheath protein FIR2 (Ge et al., 2015), all related structures contain one or two additional domains (domain III and IV) likewise organized as insertions of one another in a Russian doll arrangement. Generally, the architecture of sheath domain III and IV is less conserved and differs between the contractile systems (Supplementary Figure S4). For instance, the fold of the surface-exposed domain III of T6SSs is highly T6SS-specific and plays a crucial role for the recycling mechanism of the contracted sheath in these systems (Leiman and Shneider, 2012; Kube et al., 2014; Kudryashev et al., 2015; Brackmann et al., 2017; Wang et al., 2017).

Yet, sheath proteins containing only domains I and II still form complete sheath assemblies as has been demonstrated for FIR2 or CD1363 of the R-type pyocins and diffocins, respectively (Scholl et al., 2011; Gebhart et al., 2012; Ge et al., 2015). This indicates that the R-type bacteriocin sheath proteins represent the minimal requirement for sheath formation (Taylor et al., 2018), which is in contrast to former studies suggesting that at least three domains are necessary (Aksyuk et al., 2011 with differing domain annotation).

### Oligomeric State of the Sheath and Tube Proteins CD1363 and CD1364

The ability of the sheath and tube subunits to assemble into oligomeric structures is essential for the formation of fully functional bacteriophage tails, T6SSs or R-type PTLBs. Typically, polymerization of the tube as well as of the uncontracted sheath is initiated upon binding to initiation factors in the baseplate, and assembly of the sheath furthermore requires the tube polymer as a template (Kostyuchenko et al., 2003; Leiman and Shneider, 2012; Kudryashev et al., 2015). However, despite these external factors, preparations of purified tube and sheath proteins have been observed to also spontaneously self-assemble into oligomers *in vitro*. For instance, tube proteins of bacterial T6SSs pack into hexameric rings in the absence of baseplate components (Ballister et al., 2008; Leiman et al., 2009; Douzi et al., 2014), whereas tail sheath proteins of bacteriophage T4 as well as φKZ assemble into irreversibly contracted polysheaths of different lengths without the tube structure or the baseplate components being present (Moody, 1967; Efimov et al., 2002; Kurochkina et al., 2009). This can impede crystallization and structure determination, especially when assessing the unassembled state of these proteins.

In contrast to homologous proteins from other sources, recombinant purified R-type diffocin tube and sheath proteins CD1364 and CD1363 showed no tendency for spontaneous assembly but eluted predominantly in single prominent peaks at volumes corresponding to the molecular weight of the monomeric species in size exclusion chromatography (CD1364: 16 kDa; CD1363: 39 kDa; Supplementary Figure S1A). To further compare their oligomeric states in solution with the crystal structures described above, SAXS experiments have been performed here. As indicated by low χ-values of 0.71 for CD1364 and 1.84 for CD1363, the theoretical curves calculated from the crystal structures fitted well with the experimental SAXS data as well as with the calculated envelopes, confirming their monomeric state in solution (**Figures 2C, 3C**). Notably, in the tube protein CD1364, α1, which interacts with a neighboring molecule in the crystal structure, sticks out of the calculated envelope. This suggests that the interaction observed in the crystal structure is a consequence of crystallization and that α1 is not swapped in solution but rather packs onto the β-barrel of the same molecule such as observed for tube protein XkdM from *B. subtilis* (PDB: 2GUJ; unpublished; Supplementary Figure S2). Thus, based on our results, we can conclude that the sheath and tube proteins CD1363 and CD1364 adopt a stable monomeric pre-assembly state after translation and that this state is reflected in the crystal structures obtained here. However, as will be outlined in the following paragraph, significant structural changes are likely to accompany formation of the fully assembled R-type PTLB particle.

### Comparison to Homologs Proteins in the Free and Particle-Assembled State

As stated above, both the sheath and the tube proteins assemble to sixfold symmetrical disks that stack on top of each other with a slight right-handed rotation in PTLBs (Heymann et al., 2013; Ge et al., 2015; Nováček et al., 2016; Salih et al., 2018). Comparison of the crystal structures of the monomeric tube and sheath proteins CD1364 and CD1363 to homologous proteins in these assemblies allows for speculation about structural changes that may accompany particle formation.

**FIGURE 4:**
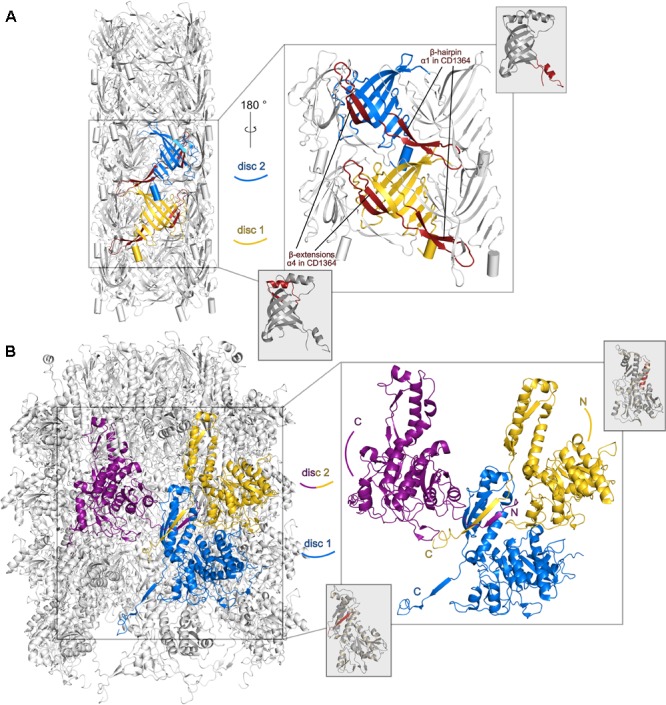
Structural differences between monomeric R-type diffocin sheath and tube proteins CD1364 and CD1363 and the respective subunits in the R-type pyocin particle. Two subunits (FIIR2) from two disks of the tube (PDB: 5W5E; Zheng et al., 2017) are shown (blue and yellow) in cartoon presentation for pyocin in **(A)**. A β-hairpin runs underneath a neighboring molecule of the same disk and establishes interactions with the molecules of one disk below. Each β-barrel is extended by two β-strands that form interactions with the adjacent central β-barrel. In **(B)**, three subunits (FIR2) from two disks of the R-type pyocin sheath assembly (PDB: 3J9Q; Ge et al., 2015) forming a four-stranded β-sheet via exchanging and refolding of the N- and C-termini are shown (labeled with N and C, respectively). For comparison, structures of the monomeric unassembled R-type diffocin tube CD1364 and sheath CD1363 proteins are presented and regions that correspond to the respective R-type pyocin elements are indicated by red coloring.

CD1364 can be superimposed with low rmsd values onto the hexameric assembly of tube protein gp104 in an electron microscopy structure of the native bacteriophage *φ812* tail (PDB: 5LI2; Nováček et al., 2016) (Supplementary Table S2). Comparison reveals that the architecture of the central β-barrel mainly remains unaltered in the assembled tube whereas positions of α-helices α2, α3, and α4 are significantly changed. In the hexameric assembly, α4 establishes interactions with neighboring subunits of the same disk and of one disk below, whereas α2 tightly packs onto the β-barrel of the same molecule (**Figure 2D** and Supplementary Figure S2). Both α-helices are located at the outer surface of the tube. Comparison with the monomeric structure of XkdM from *B. subtilis* (PDB: 2GUJ) suggests that the movement of α2 can be described as a rotation such that residues that point toward α4 in the unassembled structure face the β-barrel in the hexameric ring. Furthermore, α3 of CD1364 forms into an extended loop in gp104 and interacts with α2 of the adjacent molecule on the other site of the β-barrel. With respect to the mobile α1, no conclusions can be drawn on basis of the *φ812* tail tube structure, as the corresponding loop region is not resolved in PDB entry 5LI2. However, given the fact that the number of residues in the loop comprising α1 is almost identical even among less homologous proteins, further conclusions may be drawn from tube structures of other contractile systems, such as that of FIIR2 from R-type pyocin (PDB: 3j9Q, 5W5E; Ge et al., 2015; Zheng et al., 2017) or gp19 from the bacteriophage T4 tail (PDB: 5W5F; Zheng et al., 2017). As has been outlined above, the corresponding loop folds into a protruding β-hairpin in these assemblies (**Figure 2D** and Supplementary Figure S2). This β-hairpin runs underneath an adjacent molecule of the same disk and thus connects it to two molecules of the lower disk in these tube assemblies. Similarly, the smaller loop region that corresponds to α4 in CD1364 forms into β-strands that extend the central β-barrel in both FIIR2 and gp19 and mainly interact with the central β-sheet of the adjacent subunit (**Figure 4A**). These conformational arrangements generate a continuous antiparallel β-sheet of 24 β-strands that spans the complete inner surface of the tube structure (Ge et al., 2015; Chang et al., 2017; Wang et al., 2017; Zheng et al., 2017). Together, this indicates that assembly of the R-type diffocin tube structure not only requires movements of the four α-helices such as observed for the *φ812* tail tube but might also involve significant refolding, specifically around α1.

In the sheath protein, significant refolding upon particle assembly seems to occur within the N- and C-termini especially, as can be concluded by an overlay of monomeric CD1363 with the electron microscopy structure of FIR2 in the assembled R-type pyocin from *P. aeruginosa* (PDB: 3J9Q, 3J9R; Ge et al., 2015). While the N-terminus of monomeric CD1363 folds into a long α-helix (α1; P6–A24) that packs tightly against two other long α-helices of domain I (α9 and α10) via extensive hydrophobic interactions (**Figure 3A** magnified insert, **Figure 3B**, and Supplementary Figure S5), it adopts an extended structure in FIR2 of the *P. aeruginosa* R-type pyocin particle (**Figure 3D** and Supplementary Figure S4) to contribute a new strand to a β-sheet with three parallel and one antiparallel strand in the C-terminal region of domain I within a monomer of the next disk. This β-sheet is further expanded by a strand from the C-terminus of the monomer neighboring the chain that contributes the N-terminus from the disk below (**Figure 4B**). This creates an “interwoven mesh” that connects not only the monomers within one disk but also ties neighbored disks to one another both in the extended and contracted state (Ge et al., 2015). Similar mesh-like organizations have been found in several T6SSs, indicating that these interactions are likewise conserved in assembled R-type PTLBs (Clemens et al., 2015; Kudryashev et al., 2015; Salih et al., 2018). Interestingly, the C-terminus in monomeric CD1363 also forms a β-strand (β14) that hydrophobically interacts with residues of α9 and α10 as well as with the same residues from the neighboring β-strand (β13 in CD1363) as observed in the pyocin particle, albeit within the same chain (**Figure 3A** magnified insert and **Figure 3B**). This indicates that the length of the C-terminus is perfectly evolved to enable sheath formation by arm exchange between chains lying next to each other. At the same time, the hydrophobic character of residues in the refolded N-terminus is conserved (Supplementary Figure S5), and the finding that the N-terminus adopts a similar α-helix in the prophage sheath protein LIN1278 from *Listeria innocua* (PDB: 3LML; Aksyuk et al., 2011) suggests that the formation of the contractile apparatus of R-type PTLBs is enabled by low refolding barriers of the N- and C-terminal sequences in domain I of the sheath proteins. This is also corroborated by the crystal structure of the prophage sheath protein DSY3957 from *Desulfitobacterium hafniense* (PDB: 3HXL; Aksyuk et al., 2011), where the N-terminus displays similar structure and interactions as in the assembled FIR2 sheath structure (**Figure 3D**), even if the crystal structure does not reveal disks as in the PTLBs. Notably, sheath formation involves only residues from domain I, whereas domain II is positioned at the surface of the sheath where it does not contact other subunits or the tube (**Figure 4B**), which is also expected for domain III and IV in sheath proteins gp18, LIN1278, and DSY3957 (Supplementary Figure S4 and Supplementary Table S3).

In bacterial T6SSs as well as bacteriophage tails, baseplate gp48/54/gpU and gp25 subunits are required to initiate tube and sheath polymerization, respectively (Nakayama et al., 2000; Kostyuchenko et al., 2003; Leiman and Shneider, 2012; Brunet et al., 2015), and similar proteins are found in the gene clusters of Afp, PVC, or R-type pyocin (Sarris et al., 2014; Kube and Wendler, 2015). Using fold recognition via the Phyre2 ^7^ web server (Kelley et al., 2015), the ORF CD1370 of *C. difficile* strain 630 was found to encode a protein with high homology to the sheath initiator protein gp25 of T4 bacteriophage (PDB: 5IW9; Gebhart et al., 2012; Taylor et al., 2016), but no tube initiator could be identified via this approach. In the recent structure of the baseplate of T6SS from T4 bacteriophage (PDB: 5IV5, 5IV7), gp25 adopts the same sixfold symmetry as observed in the assembled sheath of R-type pyocins and sheath polymerization initiation likely involves a similar β-strand exchange mechanism as described above (Taylor et al., 2016, 2018). Thus, conformational changes for the first layer of pre-assembled sheath proteins are probably induced upon binding to a homologous sheath initiator protein that might be present in every baseplate complex (Brackmann et al., 2017).

### Preliminary Model of the Contractile Apparatus of R-Type Diffocin in the Extended State

**FIGURE 5:**
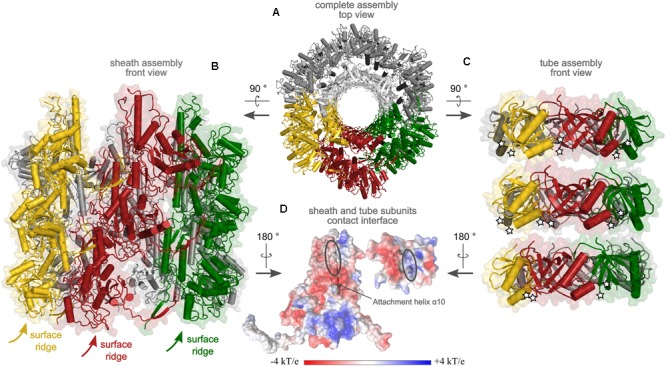
Preliminary model of the extended sheath and tube assembly of R-type diffocin, generated on the basis of the sheath from R-type pyocin (PDB: 3J9Q; Ge et al., 2015) and the tube of phage φ812 tail (PDB: 5LI2, Nováček et al., 2016). A top view onto three disks of CD1363 sheath and CD1364 tube layers is given in **(A)** with the interacting subunits colored in yellow, red, and green, respectively. In **(B)**, a side view of three disks of the CD1363 sheath assembly is shown. Because of helical disk stacking, the surface ridges wind around the particle. In **(C)**, three disks of tube subunits are presented using the same color code as in **(A,B)**. Of note, since the 5LI2 template for tube modeling contained a single tube disk only, generation of two additional disks for the R-type diffocin tube was performed via superimpositions on pyocin as described in the “Materials and Methods” section. White stars indicate positions of the attachment points for the loop comprising α-helix 1 in CD1364, which was excluded from the model due to clashes but might establish important inter-disk contacts to fill the gaps observed in the tube assembly. In **(D)**, putative contact points between sheath and tube subunits of the R-type diffocin particle with the respective electrostatic potential are shown. For CD1363, the attachment helix α10 and for CD1364, the central β-barrel, is shown as cartoon.

Based on the high homology of CD1364 to the assembled tube protein in the tail of bacteriophage *φ812* (PDB: 5LI2; Nováček et al., 2016) as well as of CD1363 to FIR2 in the assembled sheath of the extended R-type pyocin structure (PDB: 3J9Q; Ge et al., 2015), we constructed a preliminary model of the extended R-type diffocin particle using MODELLER (Webb and Šali, 2016). As common for phage tail-like contractile systems, the sheath and tube disks of the used templates are organized with identical helical symmetry (Ge et al., 2015; Taylor et al., 2016, 2018; Wang et al., 2017), and the resulting model of R-type diffocin re-iterates the observed pitch of disks in R-type pyocin (translation of approximately 38 Å, rotation of 18°; Ge et al., 2015; **Figure 5A**) However, since several residues were not resolved in the tube template (PDB: 5LI2), the corresponding loops including the potentially important region around α1 could not be modeled, causing substantial gaps between the tube disks (**Figure 5C**). In the R-type pyocin tube, these gaps are primarily occupied by the protruding β-hairpin discussed earlier, which underlines the potential importance of structural rearrangements in R-type diffocin building blocks. In the sheath assembly, in contrast, only a few residues had to be excluded and the model reflects the extensive mesh-like interactions as well as the prominent surface ridges known from other contractile systems in the extended sheath very well (**Figure 5B**) (Clemens et al., 2015; Ge et al., 2015; Kudryashev et al., 2015; Wang et al., 2017; Salih et al., 2018).

In the R-type pyocin, the main contacts between sheath and tube are established via electrostatic interactions of a positively charged region in attachment helix α10 of sheath domain I with a complementarily charged patch in the central β-barrel of the respective tube protein (Ge et al., 2015). We examined the electrostatic potential of the modeled R-type diffocin sheath and tube for similarly charged patches and our analysis indeed revealed equivalent regions in the sheath and tube but with opposite charges (**Figure 5D**). Indeed, the sequence alignment of homologous sheath proteins demonstrates that the positions of basic residues in α10 are not conserved among these proteins (Supplementary Figure S5). However, our finding of opposite charges with respect to R-type pyocin may be a consequence of our preliminary model not accurately reflecting all aspects of the diffocin particle, which is also underpinned by clashes seen between attachment helix α10 and tube residues. This reiterates once more that further rearrangements seem to be required for R-type diffocin particle formation (**Figure 5A**). The revelation of detailed interactions will thus have to await cryo-EM studies, for which promising preliminary work has recently been published (Hegarty et al., 2016).

## Conclusion

Here, we have determined the crystal structures and SAXS envelopes of the R-type diffocin tube and sheath protein CD1364 and CD1363 in their pre-assembled monomeric state. Our data indicate that several conformational changes are necessary to enable formation of the diffocin particle, which, together with the finding that recombinant monomers of both proteins were stable enough for crystallization studies, indicates that the refolding energy barriers in diffocins are higher than in similar assemblies with spontaneously self-assembling building blocks. This could make the R-type diffocins an ideal system to study the particle formation process *in vitro*. Further, the crystal structures of CD1364 and CD1363 together with cryo-EM structures of related particles enabled us to construct a preliminary model of the contractile apparatus of R-type diffocins, but because significant differences to the available templates exist, several structural details will have to be revealed in future studies addressing the complete diffocin particle.

## Author Contributions

WB designed and directed the project. NS and E-MG performed the experiments. NS, JP, and E-MG analyzed the data. NS, JP, and WB generated figures. JP wrote the manuscript with discussion and comments from all authors.

## Conflict of Interest Statement

The authors declare that the research was conducted in the absence of any commercial or financial relationships that could be construed as a potential conflict of interest.

## Footnotes

1 http://web.expasy.org/protparam/

2 http://rcsb.org/

3 http://ekhidna2.biocenter.helsinki.fi/dali/

4 http://prodata.swmed.edu/promals3d/promals3d.php

5 http://espript.ibcp.fr/ESPript/ESPript/

6 http://www.rcsb.org/pdb/workbench/workbench.do

7 http://www.sbg.bio.ic.ac.uk/phyre2/html/page.cgi?id=index

## Abbreviations

Afp: antifeeding prophage from *Serratia entomophila*
CDIs: *C. difficile* infections
Cryo-EM: cryo electron microscopy
ESI: electrospray ionization
HCP(1): hemolysin co-regulated tube protein (1)
MD: molecular dynamics
ORF: open reading frame
PDB: Protein Data Bank
PTLBs: phage tail-like bacteriocins
PVC: virulence cluster from *Photorhabdus luminescens*
Q-TOF: quadrupole time of flight
rmsd: root mean square deviations
SAD: single-wavelength anomalous dispersion
SAXS: small angle X-ray scattering
SeMet: seleno-L-methionine
SLIC: sequence- and ligation-independent-cloning
T6SS: type VI secretion system
TLS: translation liberation screw
VTFM: variable target function method

## References

[B1] Ackermann H.-W. (2003). Bacteriophage observations and evolution. *Res. Microbiol.* 154 245–251. 10.1016/S0923-2508(03)00067-6 12798228

[B2] Adams P. D. Afonine P. V. Bunkóczi G. Chen V. B. Davis I. W. Echols N. (2010). PHENIX: a comprehensive Python-based system for macromolecular structure solution. *Acta Crystallogr. D Biol. Crystallogr.* 66 213–221. 10.1107/S0907444909052925 20124702 PMC2815670

[B3] Afonine P. V. Grosse-Kunstleve R. W. Echols N. Headd J. J. Moriarty N. W. Mustyakimov M. (2012). Towards automated crystallographic structure refinement with phenix.refine. *Acta Crystallogr. D Biol. Crystallogr.* 68 352–367. 10.1107/S0907444912001308 22505256 PMC3322595

[B4] Aksyuk A. A. Kurochkina L. P. Fokine A. Forouhar F. Mesyanzhinov V. V. Tong L. (2011). Structural conservation of the Myoviridae phage tail sheath protein fold. *Structure* 19 1885–1894. 10.1016/j.str.2011.09.012 22153511 PMC3256926

[B5] Aksyuk A. A. Leiman P. G. Kurochkina L. P. Shneider M. M. Kostyuchenko V. A. Mesyanzhinov V. V. (2009). The tail sheath structure of bacteriophage T4: a molecular machine for infecting bacteria. *EMBO J.* 28 821–829. 10.1038/emboj.2009.36 19229296 PMC2670864

[B6] Baker N. A. Sept D. Joseph S. Holst M. J. McCammon J. A. (2001). Electrostatics of nanosystems: application to microtubules and the ribosome. *Proc. Natl. Acad. Sci. U.S.A.* 98 10037–10041. 10.1073/pnas.181342398 11517324 PMC56910

[B7] Ballister E. R. Lai A. H. Zuckermann R. N. Cheng Y. Mougous J. D. (2008). In vitro self-assembly of tailorable nanotubes from a simple protein building block. *Proc. Natl. Acad. Sci. U.S.A.* 105 3733–3738. 10.1073/pnas.0712247105 18310321 PMC2268831

[B8] Bartlett J. G. (1994). *Clostridium difficile*: history of its role as an enteric pathogen and the current state of knowledge about the organism. *Clin. Infect. Dis.* 18(Suppl. 4) 265–272. 10.1093/clinids/18.Supplement_4.S265 8086574

[B9] Behrens H. M. Six A. Walker D. Kleanthous C. (2017). The therapeutic potential of bacteriocins as protein antibiotics. *Emerg. Top. Life Sci.* 1 65–74. 10.1042/ETLS20160016 PMC7243282 33525816

[B10] Berman H. M. Westbrook J. Feng Z. Gilliland G. Bhat T. N. Weissig H. (2000). The protein data bank. *Nucleic Acids Res.* 28 235–242. 10.1093/nar/28.1.235 10592235 PMC102472

[B11] Berrow N. S. Alderton D. Sainsbury S. Nettleship J. Assenberg R. Rahman N. (2007). A versatile ligation-independent cloning method suitable for high-throughput expression screening applications. *Nucleic Acids Res.* 35:e45. 10.1093/nar/gkm047 17317681 PMC1874605

[B12] Bignardi G. E. (1998). Risk factors for *Clostridium difficile* infection. *J. Hosp. Infect.* 40 1–15. 10.1016/S0195-6701(98)90019-6 9777516

[B13] Bond C. S. (2003). TopDraw: a sketchpad for protein structure topology cartoons. *Bioinformatics* 19 311–312. 10.1093/bioinformatics/19.2.311 12538265

[B14] Borali E. Giacomo C. D. (2016). *Clostridium difficile* infection in children: a review. *J. Pediatr. Gastroenterol. Nutr.* 63 e130–e140. 10.1097/MPG.0000000000001264 27182626

[B15] Brackmann M. Nazarov S. Wang J. Basler M. (2017). Using force to punch holes: mechanics of contractile nanomachines. *Trends Cell Biol.* 27 623–632. 10.1016/j.tcb.2017.05.003 28602424

[B16] Brunet Y. R. Zoued A. Boyer F. Douzi B. Cascales E. (2015). The type VI secretion TssEFGK-VgrG phage-like baseplate is recruited to the TssJLM membrane complex via multiple contacts and serves as assembly platform for tail tube/sheath polymerization. *PLoS Genet.* 11:e1005545. 10.1371/journal.pgen.1005545 26460929 PMC4604203

[B17] Chang Y.-W. Rettberg L. A. Ortega D. R. Jensen G. J. (2017). In vivo structures of an intact type VI secretion system revealed by electron cryotomography. *EMBO Rep.* 18 1090–1099. 10.15252/embr.201744072 28487352 PMC5494534

[B18] Clemens D. L. Ge P. Lee B.-Y. Horwitz M. A. Zhou Z. H. (2015). Atomic structure of T6SS reveals interlaced array essential to function. *Cell* 160 940–951. 10.1016/j.cell.2015.02.005 25723168 PMC4351867

[B19] Douzi B. Spinelli S. Blangy S. Roussel A. Durand E. Brunet Y. R. (2014). Crystal structure and self-interaction of the type VI secretion tail-tube protein from enteroaggregative *Escherichia coli*. *PLoS One* 9:e86918. 10.1371/journal.pone.0086918 24551044 PMC3925092

[B20] Efimov A. V. Kurochkina L. P. Mesyanzhinov V. V. (2002). Engineering of bacteriophage T4 tail sheath protein. *Biochem. Mosc.* 67 1366–1370. 10.1023/A:1021857926152 12600265

[B21] Emsley P. Lohkamp B. Scott W. G. Cowtan K. (2010). Features and development of Coot. *Acta Crystallogr. D Biol. Crystallogr.* 66 486–501. 10.1107/S0907444910007493 20383002 PMC2852313

[B22] Evans P. R. Murshudov G. N. (2013). How good are my data and what is the resolution? *Acta Crystallogr. D Biol. Crystallogr.* 69 1204–1214. 10.1107/S0907444913000061 23793146 PMC3689523

[B23] Franke D. Svergun D. I. (2009). DAMMIF, a program for rapid ab-initio shape determination in small-angle scattering. *J. Appl. Crystallogr.* 42 342–346. 10.1107/S0021889809000338 27630371 PMC5023043

[B24] Fuchs M. R. Pradervand C. Thominet V. Schneider R. Panepucci E. Grunder M. (2014). D3, the new diffractometer for the macromolecular crystallography beamlines of the Swiss Light Source. *J. Synchrotron Radiat.* 21 340–351. 10.1107/S160057751400006X 24562555 PMC3945418

[B25] Gasteiger E. Hoogland C. Gattiker A. Duvaud S. Wilkins M. R. Appel R. D. (2005). “Protein identification and analysis tools on the ExPASy server,” in *The Proteomics Protocols Handbook* ed. Walker J. M. (New York City, NY: Humana Press) 571–607. 10.1385/1-59259-890-0:571

[B26] Ge P. Scholl D. Leiman P. G. Yu X. Miller J. F. Zhou Z. H. (2015). Atomic structures of a bactericidal contractile nanotube in its pre- and postcontraction states. *Nat. Struct. Mol. Biol.* 22 377–382. 10.1038/nsmb.2995 25822993 PMC4445970

[B27] Gebhart D. Lok S. Clare S. Tomas M. Stares M. Scholl D. (2015). A modified R-Type bacteriocin specifically targeting *Clostridium difficile* prevents colonization of mice without affecting gut microbiota diversity. *mBio* 6:e02368-14. 10.1128/mBio.02368-14 25805733 PMC4453579

[B28] Gebhart D. Williams S. R. Bishop-Lilly K. A. Govoni G. R. Willner K. M. Butani A. (2012). Novel high-molecular-weight, R-type bacteriocins of *Clostridium difficile*. *J. Bacteriol.* 194 6240–6247. 10.1128/JB.01272-12 22984261 PMC3486368

[B29] George R. H. Symonds J. M. Dimock F. Brown J. D. Arabi Y. Shinagawa N. (1978). Identification of *Clostridium difficile* as a cause of pseudomembranous colitis. *Br. Med. J.* 1:695. 10.1136/bmj.1.6114.695 630301 PMC1603073

[B30] Gerlach M. Mueller U. Weiss M. S. (2016). The MX beamlines BL14.1-3 at BESSY II. *J. Large Scale Res. Facil.* 2:A47. 10.1107/S0909049512006395 22514183 PMC3408958

[B31] Ghequire M. G. K. De Mot R. (2014). Ribosomally encoded antibacterial proteins and peptides from *Pseudomonas*. *FEMS Microbiol. Rev.* 38 523–568. 10.1111/1574-6976.12079 24923764

[B32] Ghequire M. G. K. Mot R. D. (2015). The tailocin tale: peeling off phage tails. *Trends Microbiol.* 23 587–590. 10.1016/j.tim.2015.07.011 26433692

[B33] Goldberg E. Grinius L. Letellier L. (1994). “Recognition, attachment, and injection,” in *Molecular Biology of Bacteriophage T4* ed. Karam J. D. (Washington, DC: American Society for Microbiology) 347–356.

[B34] Govan J. R. (1974). Studies on the pyocins of *Pseudomonas aeruginosa*: morphology and mode of action of contractile pyocins. *J. Gen. Microbiol.* 80 1–15. 10.1099/00221287-80-1-1 4206804

[B35] Haas H. Sacks T. Saltz N. (1974). Protective effect of pyocin against lethal *Pseudomonas aeruginosa* infections in mice. *J. Infect. Dis.* 129 470–472. 10.1093/infdis/129.4.470 4206249

[B36] Hall I. C. O’toole E. (1935). Intestinal flora in new-born infants: with a description of a new pathogenic anaerobe, *Bacillus difficilis*. *Am. J. Dis. Child.* 49 390–402. 10.1001/archpedi.1935.01970020105010

[B37] Hegarty J. P. Sangster W. Ashley R. E. Myers R. Hafenstein S. Stewart D. B. (2016). Induction and purification of *C. difficile* phage tail-like particles. *Methods Mol. Biol.* 1476 167–175. 10.1007/978-1-4939-6361-4_12 27507340

[B38] Heymann J. B. Bartho J. D. Rybakova D. Venugopal H. P. Winkler D. C. Sen A. (2013). Three-dimensional structure of the toxin-delivery particle antifeeding prophage of *Serratia entomophila*. *J. Biol. Chem.* 288 25276–25284. 10.1074/jbc.M113.456145 23857636 PMC3757192

[B39] Holm L. Laakso L. M. (2016). Dali server update. *Nucleic Acids Res.* 44 W351–W355. 10.1093/nar/gkw357 27131377 PMC4987910

[B40] Hu B. Margolin W. Molineux I. J. Liu J. (2015). Structural remodeling of bacteriophage T4 and host membranes during infection initiation. *Proc. Natl. Acad. Sci. U.S.A.* 112 E4919–E4928. 10.1073/pnas.1501064112 26283379 PMC4568249

[B41] Hurst M. R. H. Glare T. R. Jackson T. A. (2004). Cloning *Serratia entomophila* antifeeding genes–a putative defective prophage active against the grass grub *Costelytra zealandica*. *J. Bacteriol.* 186 5116–5128. 10.1128/JB.186.15.5116-5128.2004 15262948 PMC451664

[B42] Ishii S. I. Nishi Y. Egami F. (1965). The fine structure of a pyocin. *J. Mol. Biol.* 13 428–431. 10.1016/S0022-2836(65)80107-3 4956166

[B43] Kabsch W. (2010). XDS. *Acta Crystallogr. D Biol. Crystallogr.* 66 125–132. 10.1107/S0907444909047337 20124692 PMC2815665

[B44] Kabsch W. Sander C. (1983). Dictionary of protein secondary structure: pattern recognition of hydrogen-bonded and geometrical features. *Biopolymers* 22 2577–2637. 10.1002/bip.360221211 6667333

[B45] Kageyama M. (1964). Studies of a pyocin. I. Physical and chemical properties. *J. Biochem.* 55 49–53. 10.1093/oxfordjournals.jbchem.a127839 14116619

[B46] Kageyama M. Ikeda K. Egami F. (1964). Studies of a pyocin. III. Biological properties of the pyocin. *J. Biochem.* 55 59–64. 10.1093/oxfordjournals.jbchem.a127841 14116621

[B47] Kelley L. A. Mezulis S. Yates C. M. Wass M. N. Sternberg M. J. E. (2015). The Phyre2 web portal for protein modeling, prediction and analysis. *Nat. Protoc.* 10 845–858. 10.1038/nprot.2015.053 25950237 PMC5298202

[B48] Kocíncová D. Lam J. S. (2013). A deletion in the wapB promoter in many serotypes of *Pseudomonas aeruginosa* accounts for the lack of a terminal glucose residue in the core oligosaccharide and resistance to killing by R3-pyocin. *Mol. Microbiol.* 89 464–478. 10.1111/mmi.12289 23750877

[B49] Köhler T. Donner V. Delden C. V. (2010). Lipopolysaccharide as shield and receptor for R-pyocin-mediated killing in *Pseudomonas aeruginosa*. *J. Bacteriol.* 192 1921–1928. 10.1128/JB.01459-09 20118263 PMC2838038

[B50] Konarev P. V. Volkov V. V. Sokolova A. V. Koch M. H. J. Svergun D. I. (2003). PRIMUS: a windows PC-based system for small-angle scattering data analysis. *J. Appl. Crystallogr.* 36 1277–1282. 10.1107/S0021889803012779

[B51] Kostyuchenko V. A. Leiman P. G. Chipman P. R. Kanamaru S. van Raaij M. J. Arisaka F. (2003). Three-dimensional structure of bacteriophage T4 baseplate. *Nat. Struct. Mol. Biol.* 10 688–693. 10.1038/nsb970 12923574

[B52] Kube S. Wendler P. (2015). Structural comparison of contractile nanomachines. *Biophysics* 2 88–115. 10.3934/biophy.2015.2.88

[B53] Kudryashev M. Wang R. Y.-R. Brackmann M. Scherer S. Maier T. Baker D. (2015). Structure of the type VI secretion system contractile sheath. *Cell* 160 952–962. 10.1016/j.cell.2015.01.037 25723169 PMC4359589

[B54] Kurochkina L. P. Aksyuk A. A. Sachkova M. Y. Sykilinda N. N. Mesyanzhinov V. V. (2009). Characterization of tail sheath protein of giant bacteriophage φKZ *Pseudomonas aeruginosa*. *Virology* 395 312–317. 10.1016/j.virol.2009.09.015 19822340

[B55] Kuroda K. Kageyama M. (1979). Biochemical properties of a new flexuous bacteriocin, pyocin F1, produced by *Pseudomonas aeruginosa*. *J. Biochem.* 85 7–19. 10.1093/oxfordjournals.jbchem.a132332 104991

[B56] Larson H. E. Parry J. V. Price A. B. Davies D. R. Dolby J. Tyrrell D. A. (1977). Undescribed toxin in pseudomembranous colitis. *Br. Med. J.* 1 1246–1248. 10.1136/bmj.1.6071.1246 861560 PMC1607118

[B57] Lee G. Chakraborty U. Gebhart D. Govoni G. R. Zhou Z. H. Scholl D. (2016). F-type bacteriocins of *Listeria monocytogenes*: a new class of phage tail-like structures reveals broad parallel coevolution between tailed bacteriophages and high-molecular-weight bacteriocins. *J. Bacteriol.* 198 2784–2793. 10.1128/JB.00489-16 27457717 PMC5038007

[B58] Leiman P. G. Basler M. Ramagopal U. A. Bonanno J. B. Sauder J. M. Pukatzki S. (2009). Type VI secretion apparatus and phage tail-associated protein complexes share a common evolutionary origin. *Proc. Natl. Acad. Sci. U.S.A.* 106 4154–4159. 10.1073/pnas.0813360106 19251641 PMC2657435

[B59] Leiman P. G. Shneider M. M. (2012). “Contractile tail machines of bacteriophages,” in *Viral Molecular Machines* eds Rossmann M. G. Rao V. B. (Boston, MA: Springer) 93–114. 10.1007/978-1-4614-0980-9_5 22297511

[B60] Lessa F. C. Mu Y. Bamberg W. M. Beldavs Z. G. Dumyati G. K. Dunn J. R. (2015). Burden of *Clostridium difficile* infection in the United States. *N. Engl. J. Med.* 372 825–834. 10.1056/NEJMoa1408913 25714160 PMC10966662

[B61] Li M. Z. Elledge S. J. (2007). Harnessing homologous recombination in vitro to generate recombinant DNA via SLIC. *Nat. Methods* 4 251–256. 10.1038/nmeth1010 17293868

[B62] Lim Y. T. Jobichen C. Wong J. Limmathurotsakul D. Li S. Chen Y. (2015). Extended loop region of Hcp1 is critical for the assembly and function of type VI secretion system in *Burkholderia pseudomallei*. *Sci. Rep.* 5:8235. 10.1038/srep08235 25648885 PMC4650826

[B63] Matsui H. Sano Y. Ishihara H. Shinomiya T. (1993). Regulation of pyocin genes in *Pseudomonas aeruginosa* by positive (prtN) and negative (prtR) regulatory genes. *J. Bacteriol.* 175 1257–1263. 10.1128/jb.175.5.1257-1263.1993 8444788 PMC193209

[B64] Matthews B. W. (1968). Solvent content of protein crystals. *J. Mol. Biol.* 33 491–497. 10.1016/0022-2836(68)90205-2 5700707

[B65] McCoy A. J. Grosse-Kunstleve R. W. Adams P. D. Winn M. D. Storoni L. C. Read R. J. (2007). Phaser crystallographic software. *J. Appl. Crystallogr.* 40 658–674. 10.1107/S0021889807021206 19461840 PMC2483472

[B66] Meadow P. M. Wells P. L. (1978). Receptor sites for R-type pyocins and bacteriophage E79 in the core part of the lipopolysaccharide of *Pseudomonas aeruginosa* PAC1. *Microbiology* 108 339–343. 10.1099/00221287-108-2-339

[B67] Merrikin D. J. Terry C. S. (1972). Use of pyocin 78-C2 in the treatment of *Pseudomonas aeruginosa* infection in mice. *Appl. Microbiol.* 23 164–165. 4621792 10.1128/am.23.1.164-165.1972 PMC380297

[B68] Michel-Briand Y. Baysse C. (2002). The pyocins of *Pseudomonas aeruginosa*. *Biochimie* 84 499–510. 10.1016/S0300-9084(02)01422-0 12423794

[B69] Miller J. H. (1972). *Experiments in Molecular Genetics.* Cold Spring Harbor, NY: Cold Spring Harbor Laboratory.

[B70] Moody M. F. (1967). Structure of the sheath of bacteriophage T4: I. Structure of the contracted sheath and polysheath. *J. Mol. Biol.* 25 167–200. 10.1016/0022-2836(67)90136-2 6034098

[B71] Nakayama K. Takashima K. Ishihara H. Shinomiya T. Kageyama M. Kanaya S. (2000). The R-type pyocin of *Pseudomonas aeruginosa* is related to P2 phage, and the F-type is related to lambda phage. *Mol. Microbiol.* 38 213–231. 10.1046/j.1365-2958.2000.02135.x 11069649

[B72] Nováček J. Šiborová M. Benešík M. Pantůček R. Doškař J. Plevka P. (2016). Structure and genome release of Twort-like Myoviridae phage with a double-layered baseplate. *Proc. Natl. Acad. Sci. U.S.A.* 113 9351–9356. 10.1073/pnas.1605883113 27469164 PMC4995954

[B73] Pei J. Kim B.-H. Grishin N. V. (2008). PROMALS3D: a tool for multiple protein sequence and structure alignments. *Nucleic Acids Res.* 36 2295–2300. 10.1093/nar/gkn072 18287115 PMC2367709

[B74] Pernot P. Round A. Barrett R. De Maria Antolinos A. Gobbo A. Gordon E. (2013). Upgraded ESRF BM29 beamline for SAXS on macromolecules in solution. *J. Synchrotron Radiat.* 20 660–664. 10.1107/S0909049513010431 23765312 PMC3943554

[B75] Pettersen E. F. Goddard T. D. Huang C. C. Couch G. S. Greenblatt D. M. Meng E. C. (2004). UCSF Chimera–a visualization system for exploratory research and analysis. *J. Comput. Chem.* 25 1605–1612. 10.1002/jcc.20084 15264254

[B76] Poutanen S. M. Simor A. E. (2004). *Clostridium difficile*-associated diarrhea in adults. *CMAJ* 171 51–58. 10.1503/cmaj.1031189 15238498 PMC437686

[B77] Ritchie J. M. Greenwich J. L. Davis B. M. Bronson R. T. Gebhart D. Williams S. R. (2011). An *Escherichia coli* O157-specific engineered pyocin prevents and ameliorates infection by *E. coli* O157:H7 in an animal model of diarrheal disease. *Antimicrob. Agents Chemother.* 55 5469–5474. 10.1128/AAC.05031-11 21947394 PMC3232761

[B78] Robert X. Gouet P. (2014). Deciphering key features in protein structures with the new ENDscript server. *Nucleic Acids Res.* 42 W320–W324. 10.1093/nar/gku316 24753421 PMC4086106

[B79] Šali A. Blundell T. L. (1993). Comparative protein modelling by satisfaction of spatial restraints. *J. Mol. Biol.* 234 779–815. 10.1006/jmbi.1993.1626 8254673

[B80] Salih O. He S. Planamente S. Stach L. MacDonald J. T. Manoli E. (2018). Atomic structure of type VI contractile sheath from *Pseudomonas aeruginosa*. *Structure* 26 329.e3–336.e3. 10.1016/j.str.2017.12.005 29307484 PMC5807055

[B81] Sarris P. F. Ladoukakis E. D. Panopoulos N. J. Scoulica E. V. (2014). A Phage tail-derived element with wide distribution among both prokaryotic domains: a comparative genomic and phylogenetic study. *Genome Biol. Evol.* 6 1739–1747. 10.1093/gbe/evu136 25015235 PMC4122934

[B82] Scholl D. (2017). Phage tail-like bacteriocins. *Annu. Rev. Virol.* 4 453–467. 10.1146/annurev-virology-101416-041632 28961412

[B83] Scholl D. Cooley M. Williams S. R. Gebhart D. Martin D. Bates A. (2009). An engineered R-type pyocin is a highly specific and sensitive bactericidal agent for the food-borne pathogen *Escherichia coli* O157:H7. *Antimicrob. Agents Chemother.* 53 3074–3080. 10.1128/AAC.01660-08 19349519 PMC2704633

[B84] Scholl D. Gebhart D. Williams S. R. Bates A. Mandrell R. (2012). Genome sequence of *E. coli* O104:H4 leads to rapid development of a targeted antimicrobial agent against this emerging pathogen. *PLoS One* 7:e33637. 10.1371/journal.pone.0033637 22432037 PMC3303846

[B85] Scholl D. Gebhart D. Williams S. R. Govoni G. R. Martin D. (2011). Diffocin and methods of use thereof. U.S. Patent No. 8673291. Washington, DC: U.S. Patent and Trademark Office.

[B86] Scholl D. Martin D. W. (2008). Antibacterial efficacy of R-type pyocins towards *Pseudomonas aeruginosa* in a murine peritonitis model. *Antimicrob. Agents Chemother.* 52 1647–1652. 10.1128/AAC.01479-07 18332164 PMC2346647

[B87] Schrödinger LLC (2010). *The PyMOL Molecular Graphics System Version 1. 8. 2. 3.* New York City, NY: Schrödinger, LLC.

[B88] Sharp R. (2001). Bacteriophages: biology and history. *J. Chem. Technol. Biotechnol.* 76 667–672. 10.1002/jctb.434

[B89] Slimings C. Riley T. V. (2014). Antibiotics and hospital-acquired *Clostridium difficile* infection: update of systematic review and meta-analysis. *J. Antimicrob. Chemother.* 69 881–891. 10.1093/jac/dkt477 24324224

[B90] Strauch E. Kaspar H. Schaudinn C. Dersch P. Madela K. Gewinner C. (2001). Characterization of enterocoliticin, a phage tail-like bacteriocin, and its effect on pathogenic *Yersinia enterocolitica* strains. *Appl. Environ. Microbiol.* 67 5634–5642. 10.1128/AEM.67.12.5634-5642.2001 11722917 PMC93354

[B91] Kube S. Kapitein N. Zimniak T. Herzog F. Mogk A. Wendler P. (2014). Structure of the VipA/B type VI secretion complex suggests a contraction-state-specific recycling mechanism. *Cell Rep.* 8 20–30. 10.1016/j.celrep.2014.05.034 24953649

[B92] Studier F. W. (2014). Stable expression clones and auto-induction for protein production in *E. coli*. *Methods Mol. Biol.* 1091 17–32. 10.1007/978-1-62703-691-7_2 24203322

[B93] Svergun D. Barberato C. Koch M. H. J. (1995). CRYSOL – a program to evaluate X-ray solution scattering of biological macromolecules from atomic coordinates. *J. Appl. Crystallogr.* 28 768–773. 10.1107/S0021889895007047

[B94] Takeda Y. Kageyama M. (1975). Subunit arrangement in the extended sheath of pyocin R. *J. Biochem.* 77 679–684. 10.1093/oxfordjournals.jbchem.a130770 807567

[B95] Takeya K. Mlnamishima Y. Amako K. Ohnishi Y. (1967). A small rod-shaped pyocin. *Virology* 31 166–168. 10.1016/0042-6822(67)90021-9 4959705

[B96] Taylor N. M. I. Prokhorov N. S. Guerrero-Ferreira R. C. Shneider M. M. Browning C. Goldie K. N. (2016). Structure of the T4 baseplate and its function in triggering sheath contraction. *Nature* 533 346–352. 10.1038/nature17971 27193680

[B97] Taylor N. M. I. van Raaij M. J. Leiman P. G. (2018). Contractile injection systems of bacteriophages and related systems. *Mol. Microbiol.* 108 6–15. 10.1111/mmi.13921 29405518

[B98] Tenover F. C. Tickler I. A. Persing D. H. (2012). Antimicrobial-resistant strains of *Clostridium difficile* from North America. *Antimicrob. Agents Chemother.* 56 2929–2932. 10.1128/AAC.00220-12 22411613 PMC3370774

[B99] Terwilliger T. C. Adams P. D. Read R. J. McCoy A. J. Moriarty N. W. Grosse-Kunstleve R. W. (2009). Decision-making in structure solution using Bayesian estimates of map quality: the PHENIX AutoSol wizard. *Acta Crystallogr. D Biol. Crystallogr.* 65 582–601. 10.1107/S0907444909012098 19465773 PMC2685735

[B100] Terwilliger T. C. Grosse-Kunstleve R. W. Afonine P. V. Moriarty N. W. Zwart P. H. Hung L. W. (2008). Iterative model building, structure refinement and density modification with the PHENIX AutoBuild wizard. *Acta Crystallogr. D Biol. Crystallogr.* 64 61–69. 10.1107/S090744490705024X 18094468 PMC2394820

[B101] Theriot C. M. Young V. B. (2015). Interactions between the gastrointestinal microbiome and *Clostridium difficile*. *Annu. Rev. Microbiol.* 69 445–461. 10.1146/annurev-micro-091014-104115 26488281 PMC4892173

[B102] Touw W. G. Baakman C. Black J. te Beek T. A. H. Krieger E. Joosten R. P. (2015). A series of PDB-related databanks for everyday needs. *Nucleic Acids Res.* 43 D364–D368. 10.1093/nar/gku1028 25352545 PMC4383885

[B103] Uratani Y. Hoshino T. (1984). Pyocin R1 inhibits active transport in *Pseudomonas aeruginosa* and depolarizes membrane potential. *J. Bacteriol.* 157 632–636. 6420392 10.1128/jb.157.2.632-636.1984 PMC215293

[B104] Veesler D. Cambillau C. (2011). A common evolutionary origin for tailed-bacteriophage functional modules and bacterial machineries. *Microbiol. Mol. Biol. Rev.* 75 423–433. 10.1128/MMBR.00014-11 21885679 PMC3165541

[B105] Vohra P. Poxton I. R. (2011). Efficacy of decontaminants and disinfectants against *Clostridium difficile*. *J. Med. Microbiol.* 60 1218–1224. 10.1099/jmm.0.030288-0 21474613

[B106] Wang J. Brackmann M. Castaño-Díez D. Kudryashev M. Goldie K. N. Maier T. (2017). Cryo-EM structure of the extended type VI secretion system sheath-tube complex. *Nat. Microbiol.* 2 1507–1512. 10.1038/s41564-017-0020-7 28947741

[B107] Webb B. Šali A. (2016). Comparative protein structure modeling using MODELLER. *Curr. Protoc. Bioinformatics* 54 5.6.1–5.6.37. 10.1002/cpbi.3 27322406 PMC5031415

[B108] Williams S. R. Gebhart D. Martin D. W. Scholl D. (2008). Retargeting R-type pyocins to generate novel bactericidal protein complexes. *Appl. Environ. Microbiol.* 74 3868–3876. 10.1128/AEM.00141-08 18441117 PMC2446544

[B109] Winn M. D. Ballard C. C. Cowtan K. D. Dodson E. J. Emsley P. Evans P. R. (2011). Overview of the CCP4 suite and current developments. *Acta Crystallogr. D Biol. Crystallogr.* 67 235–242. 10.1107/S0907444910045749 21460441 PMC3069738

[B110] Yang G. Dowling A. J. Gerike U. ffrench-Constant R. H. Waterfield N. R. (2006). Photorhabdus virulence cassettes confer injectable insecticidal activity against the wax moth. *J. Bacteriol.* 188 2254–2261. 10.1128/JB.188.6.2254-2261.2006 16513755 PMC1428146

[B111] Zheng W. Wang F. Taylor N. M. I. Guerrero-Ferreira R. C. Leiman P. G. Egelman E. H. (2017). Refined Cryo-EM structure of the T4 tail tube: exploring the lowest dose limit. *Structure* 25 1436.e2–1441.e2. 10.1016/j.str.2017.06.017 28757144 PMC5587399

[B112] Zilberberg M. D. Shorr A. F. Kollef M. H. (2008). Increase in adult *Clostridium difficile*-related hospitalizations and case-fatality rate, United States, 2000-2005. *Emerg. Infect. Dis.* 14 929–931. 10.3201/eid1406.071447 18507904 PMC2600276

