# Supplementary material for: Crystal Structures of R-Type Bacteriocin Sheath and Tube Proteins CD1363 and CD1364 From Clostridium difficile in the Pre-assembled State
Source: Front Microbiol. 2018 Aug 3;9:1750. doi: 10.3389/fmicb.2018.01750 (PMC6088184; doi:10.3389/fmicb.2018.01750)
Supplement: Supplementary file 1 [file Presentation_1.pdf]

Supplementary Material

Crystal structures of R-type bacteriocin sheath and tube proteins CD1363 and CD1364 from *Clostridium difficile* in the pre-assembled state

Nina Schwemmlein, Jan Pippel, Emerich-Mihai Gazdag & Wulf Blankenfeldt*

*** Correspondence:** Corresponding Author: wulf.blankenfeldt@helmholtz-hzi.de

1 Supplementary Figures and Tables

1.1 Supplementary Figures

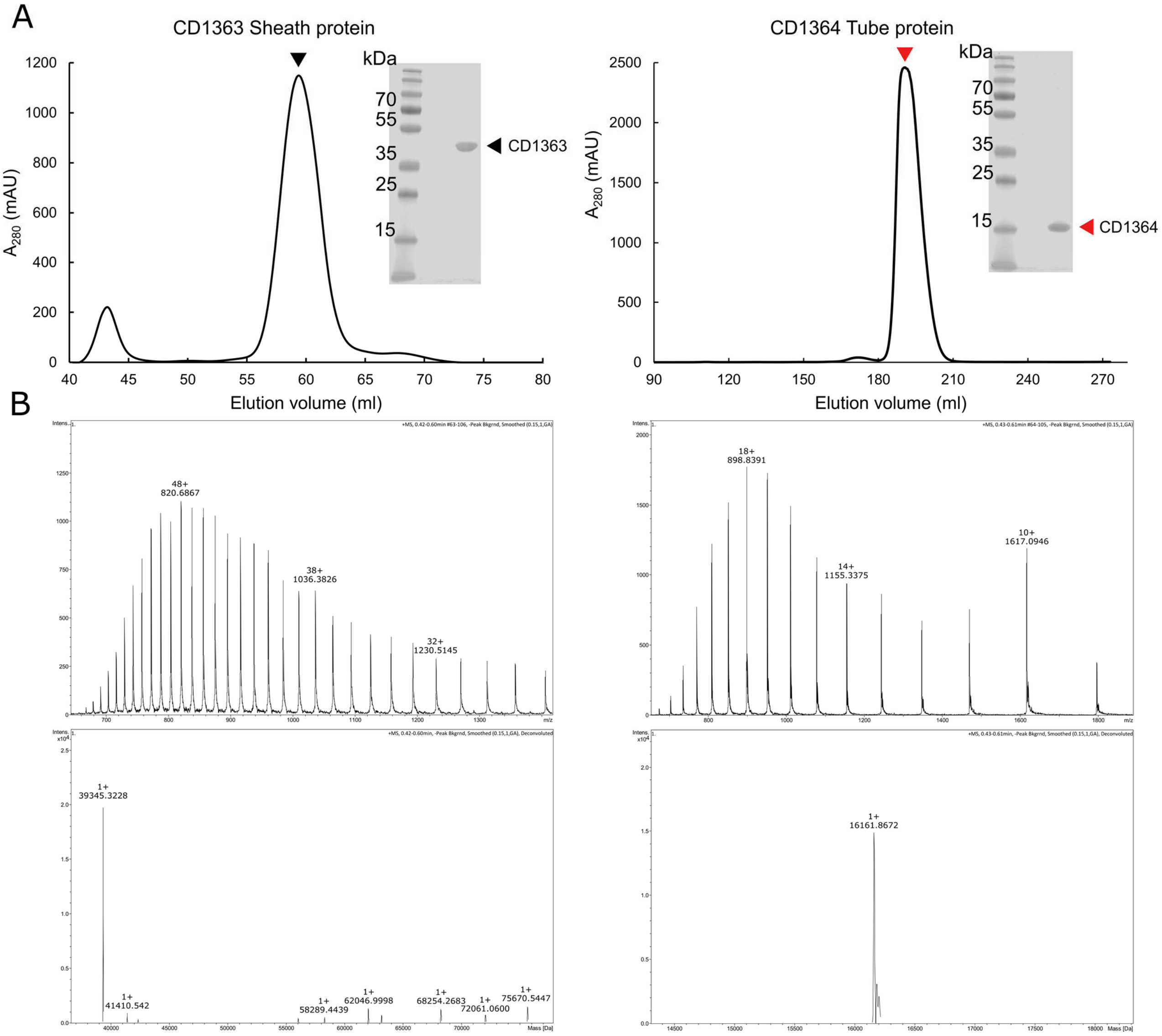

Figure S1: Analysis of the purified R-type diffocin sheath and tube proteins CD1363 and CD1364. In **A,** chromatograms of the final gel filtration steps with corresponding SDS-PAGE gels of the pooled fractions for the indicated peaks are shown. CD1363 (39 kDa) and CD1364 (16 kDa) were purified via a Superdex 75 16/60 and a Superdex 75 26/60 prep grade column (column volumes: 120/320 mL; both GE Healthcare Life Sciences), respectively. In **B**, acquired ESI-TOF mass spectra are shown, displaying multiply charged ions from +59 to +28 for CD1363 (upper left panel) and from +22 to +9 for CD1364 (upper right panel) and exemplary mass peaks were labeled. The spectra were smoothed and deconvoluted to obtain singly charged spectra (lower right and left panel) with mass peaks that correspond to the theoretical molecular weights for CD1363 and CD1364.

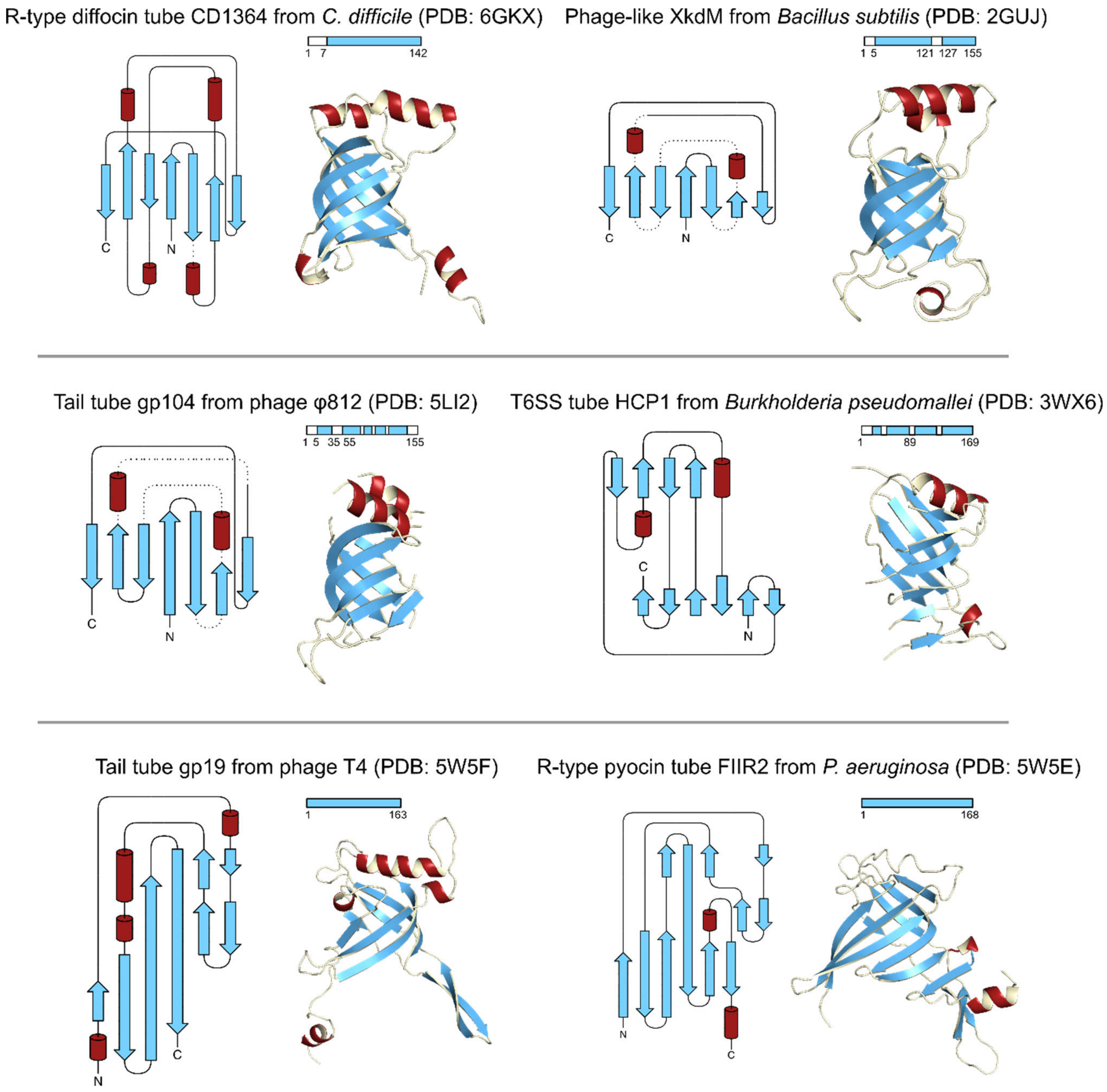

Figure S2: Structural organization of tube protein homologs of CD1364 from R-type diffocin. The crystal structures and the corresponding topology diagrams of R-type diffocin tube protein CD1364 from *Clostridium difficile* (PDB: 6GKX; this study), XkdM tube protein from *Bacillus subtilis* (PDB: 2GUJ; unpublished), tube protein gp104 from bacteriophage φ812 (PDB: 5LI2; Nováček et al., 2016), T6SS tube protein from *Burkholderia pseudomallei* (PDB: 3WX6; Lim et al., 2015), HCP1 from *Flavobacterium johnsoniae* (PDB: 6BDC, unpublished), gp19 from the bacteriophage T4 tail (PDB: 5W5F) and R-type pyocin tube protein FIIR2 from *Pseudomonas aeruginosa* (PDB: 5W5E; both Zheng et al., 2017) are shown. The central β-sheet and α-helices are colored in blue and red, respectively. White regions in the panels above the crystal structures indicate unresolved areas in the structure.

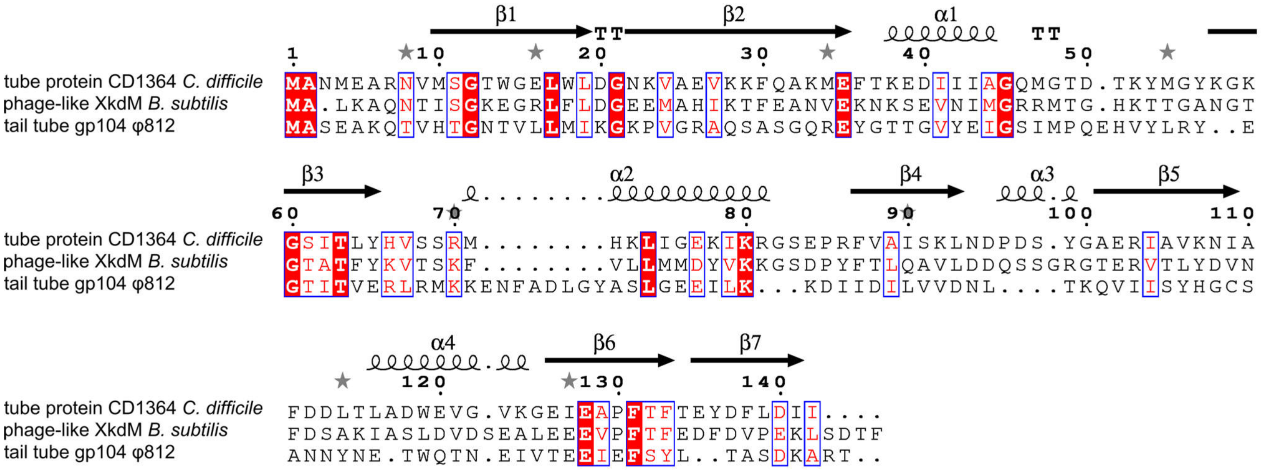

Figure S3: Sequence alignment of CD1364 with homologous tube proteins. Secondary structure assignment and sequence numbering corresponds to the structure of CD1364.

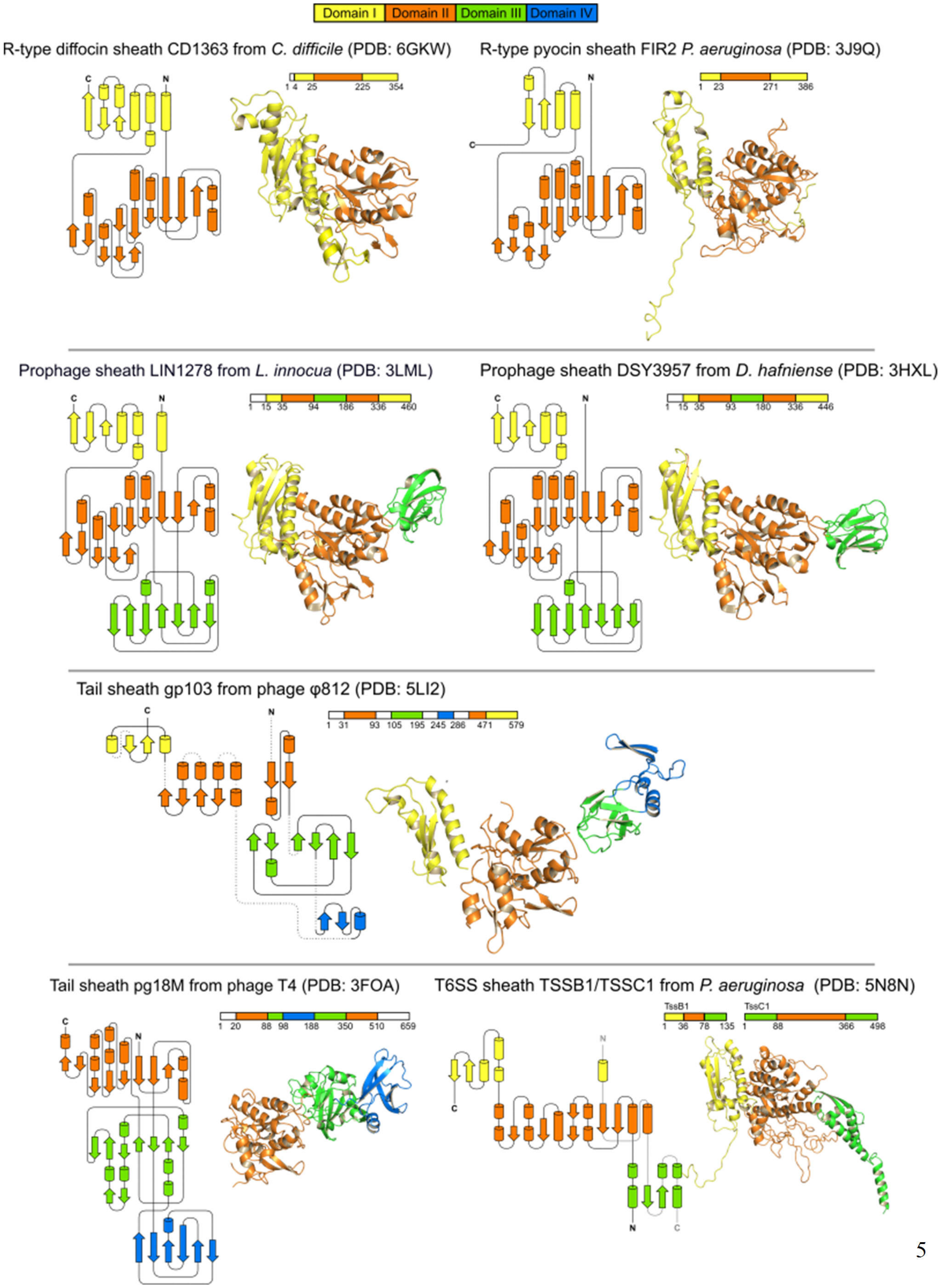
Domain I
Domain II
Domain III
Domain IV
R-type diffocin sheath CD1363 from C. difficile (PDB: 6GKW)
1 4 25 225 354
R-type pyocin sheath FIR2 P. aeruginosa (PDB: 3J9Q)
1 23 271 386
Prophage sheath LIN1278 from L. innocua (PDB: 3LML)
1 15 35 94 186 336 460
Prophage sheath DSY3957 from D. hafniense (PDB: 3HXL)
1 15 35 93 180 336 446
Tail sheath gp103 from phage φ812 (PDB: 5LI2)
1 31 93 105 195 245 286 471 579
Tail sheath pg18M from phage T4 (PDB: 3FOA)
1 20 88 98 188 350 510 659
T6SS sheath TSSB1/TSSC1 from P. aeruginosa (PDB: 5N8N)
TssB1
1 36 78 135
TssC1
1 88 366 498

Figure S4: Domain composition of the R-type diffocin sheath CD1363 and of homologous proteins. Crystal structures and topology diagrams of R-type diffocin sheath protein CD1363 from *Clostridium difficile* (PDB: 6GKW; this study), R-type pyocin sheath protein FIR2 from *Pseudomonas aeruginosa* (PDB: 3J9Q; Ge et al., 2015), prophage sheath protein LIN1278 from *Listeria innocua* (PDB: 3LML), prophage sheath protein DSY3957 from *Desulfitobacterium hafniense* (PDB: 3HXL; both Aksyuk et al., 2011), tail sheath protein gp103 from phage φ812 (PDB: 5LI2; Nováček et al., 2016), the sheath protein protease-resistant mutant fragment gp18M from phage T4 (PDB: 3FOA; Aksyuk et al., 2009), and the T6SS sheath protein complex TSSB1/TSSC1 from *Pseudomonas aeruginosa* (PDB: 5N8N; Salih et al., 2018) are shown. Domain boundaries are depicted in the panels above the respective crystal structures with colors according to the central color scheme shown in the top panel (white regions illustrate parts of the protein sequence that are not resolved in the protein structure). The figure was adapted and extended from Aksyuk et al., 2009, 2011 and Leiman and Shneider, 2012.

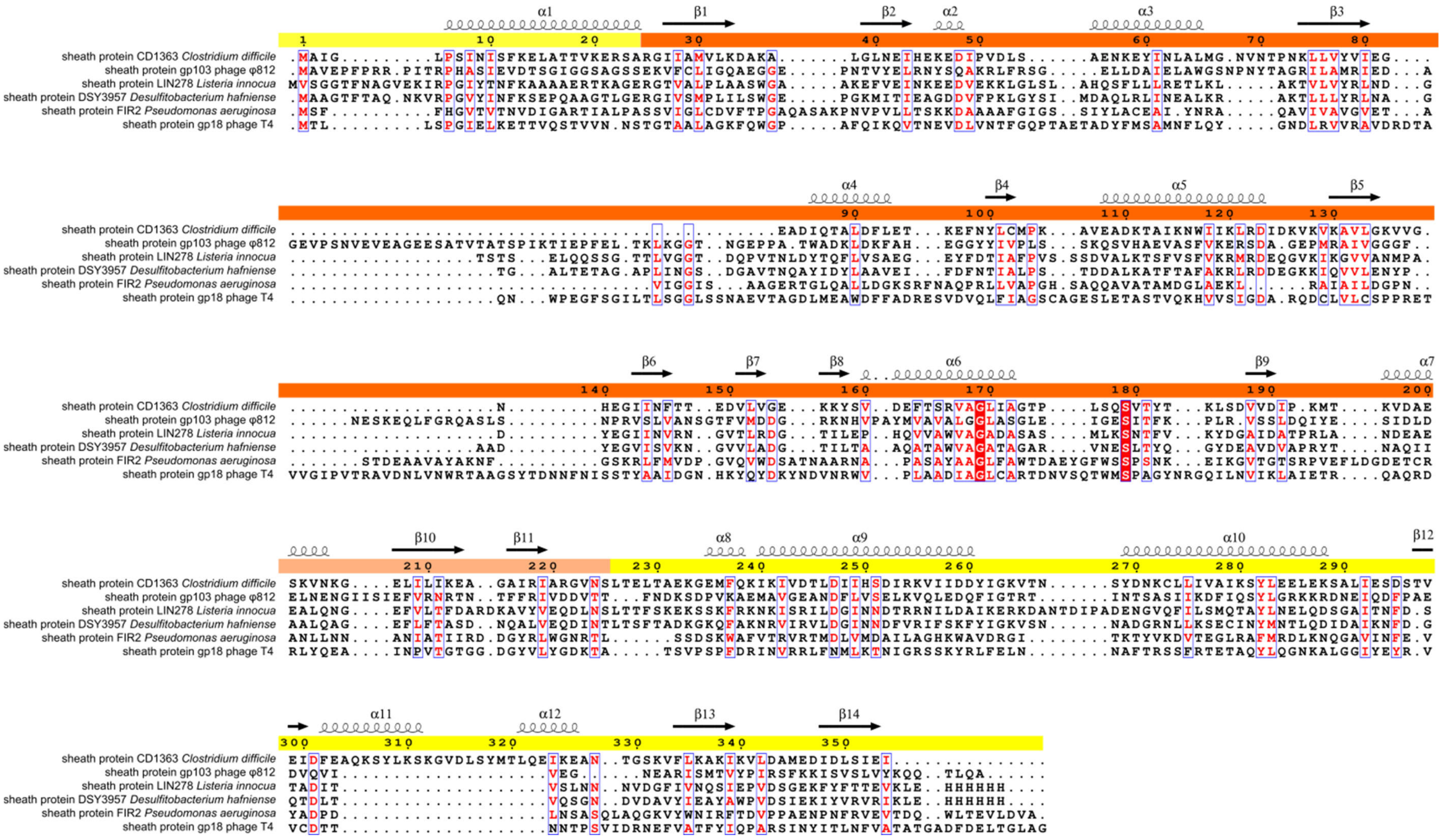

Figure S5: Sequence alignment of CD1363 with homologous sheath proteins. Secondary structure assignment and sequence numbering corresponds to the structure of CD1363. Color code for domain I (yellow) and II (orange) is identical to Figure 2. Domains III and/or IV, which are not present in CD1363 were excluded from the sequences of gp103 and gp18 from phage φ812 and phage T4 tail sheath proteins.

1.2 Supplementary Tables

Table S1: Overview of oligonucleotide primers used for SLIC cloning.

Oligonucleotide	Sequence 5’ → 3’
pOPIN-His-MBP-CD1363_fwd	GAAGTTCTGTTTCAGGGTCCCATGGCTATAGGATTACCAAGTATCAACATATCATTTAAG
pOPIN-His-MBP-CD1363_rev	TAAACTGGTCTAGAAAGCTTATATTTCTATTGATAAATCTATATCTTCCATAGCATCAAGTACTTTTATTTTTG
pOPIN-His-MBP-CD1364_fwd	GAAGTTCTGTTTCAGGGTCCCATGGCAAATATGGAAGCTAGAAATGTAATGAGT
pOPIN-His-MBP-CD1364_rev	TAAACTGGTCTAGAAAGCTTAAATTATATCAAGAAAATCATACTCAGTAAATGTGAAAGGTGC

Table S2: Summary of homologous crystal structures from tube proteins with root mean square deviations (rmsd), Z-Scores and sequence identities towards *Clostridium difficile* tube protein CD1364.

Protein name	Source organism	PDB-code	Z-Score	Rmsd [Å]	Sequence identity [%]	Reference
Phage-like element PBSX protein XkdM (pre-assembled)	*Bacillus subtilis*	2GUJ	12.2	2.9	34	-
Phage tail tube protein gp104* (native tail)	Bacteriophage φ812*	5LI2	8.3	2.4	33*/18	Nováček et al., 2016
Type VI secretion system tube haemolysin co-regulated protein 1 (HCP1) (hexamer)	*Burkholderia pseudomallei*	3WX6	5.3	4.1	8	Lim et al., 2015
Phage tail tube protein gp19 (assembled tube)	Bacteriophage T4	5W5F	4.8	4.4	11	Zheng et al., 2017
Type VI secretion system tube Haemolysin co-regulated protein 1 (HCP1) (hexamer)	*Flavobacterium johnsoniae*	6BDC	4.7	5.5	9	-
Type VI secretion system tube Haemolysin co-regulated protein 1 (HCP1) (hexamer)	*Pseudomonas aeruginosa*	1Y12	4.7	5.2	12	Mougous et al., 2006
Phage tail tube protein pb6 (trimer)	Bacteriophage T5	5NGJ	4.4	4.0	8	Arnaud et al., 2017
Type VI secretion system tube haemolysin co-regulated protein 2 (HCP2) (hexamer)	*Salmonella typhimurium*	5XEU	4.1	3.1	11	Lin et al., 2017
Type VI secretion system tube haemolysin co-regulated protein (HCP) (assembled tube)	*Vibrio cholerae*	5MXN	4.0	4.9	9	Wang et al., 2017
N-terminal domain of phage tail tube protein gpV (solution structure)	Bacteriophage λ	2K4Q	4.0	3.6	13	Pell et al., 2009
Type VI secretion system tube haemolysin co-regulated protein (HCP) (assembled tube)	*Myxococcus xanthus*	5URW	4.0	5.3	9	Chang et al., 2017

R-type pyocin tube protein FIIR2 (assembled tube)	*Pseudomonas aeruginosa*	5W5E	3.3	3.9	8	Zheng et al., 2017

* sequence of fitted model is identical to PDB: 2GUJ

Table S3: Summary of crystal structures from sheath proteins homologous to *Clostridium difficile* sheath protein CD1363 with root mean square deviations (rmsd), Z-Scores, sequence identities and according domain composition.

Protein name (state)	Source organism	PDB-code	Z-Score	Rmsd [Å]	Sequence identity [%]	Domains	Reference
Prophage sheath protein LIN1278 (unassembled)	*Listeria innocua*	3LML	36.5	2.4	26	I, II, III	Aksyuk et al., 2011
Prophage sheath protein DSY3957 (unassembled)	*Desulfitobacterium hafniense*	3HXL	33.6	2.4	25	I, II, III	Aksyuk et al., 2011
R-type pyocin sheath protein FIR2 (contracted)	*Pseudomonas aeruginosa*	3J9Q	23.6	2.8	14	I, II	Ge et al., 2015
R-type pyocin sheath protein FIR2 (extended)	*Pseudomonas aeruginosa*	3J9R	23.5	3.1	14	I, II	Ge et al., 2015
Modeled full-length T4 phage tail sheath protein gp18 (extended)	Bacteriophage T4	3J2M	23.2	2.4	11	I, II, III, IV*	Fokine et al., 2013
Modeled full-length T4 phage tail sheath protein gp18 (contracted)	Bacteriophage T4	3J2N	19.4	2.4	11	I, II, III, IV*	Fokine et al., 2013
Phage tail sheath protein gp103 (extended)	Bacteriophage φ812	5LI2	19.2	3.4	11	I, II, III, IV	Nováček et al., 2016
Type VI secretion system sheath assembly TssB1/TssC1 (contracted)	*Pseudomonas aeruginosa*	5N8N	19.0	3.1	7	I, II, III**	Salih et al., 2018

Phage tail sheath protein gp103 (contracted)	Bacteriophage φ812	5LI4	18.7	2.9	11	I, II, III, IV	Nováček et al., 2016
Type VI secretion system sheath assembly IglA/IglB (contracted)	*Francisella tularensis*	3J9O	18.4	3.2	8	I, II, III**	Clemens et al., 2015
Type VI secretion system sheath assembly VipA-N2/VipB† (contracted)	*Vibrio cholerae*	5MYU	16.2	3.2	7	I, II, III**	Wang et al., 2017
Type IV secretion system sheath assembly TssB/TssC (extended)	*Myxococcus xanthus*	5URW	15.8	3.0	5	I, II, III**	Chang et al., 2017
Type IV secretion system sheath assembly VipA/VipB (contracted)	*Vibrio cholerae*	3J9G	15.8	3.1	7	I, II, III**	Kudryashev et al., 2015
Type VI secretion system sheath assembly VipA-N3/VipB† (extended)	*Vibrio cholerae*	5MXN	15.7	3.2	6	I, II, III**	Wang et al., 2017
Type VI secretion system sheath assembly TssB/TssC (contracted)	*Myxococcus xanthus*	5URX	15.2	3.1	6	I, II, III**	Chang et al., 2017
protease-resistant fragment of T4 phage tail sheath protein mutant gp18M (unassembled)	Bacteriophage T4	3FOA	12.7	2.4	12	II, III, IV***	Aksyuk et al., 2009

protease-resistant fragment of T4 phage tail sheath protein mutant gp18M (contracted)	Bacteriophage T4	3FOI	12.7	2.4	12	II, III, IV***	Aksyuk et al., 2009
protease-resistant fragment of T4 phage tail sheath protein mutant gp18M (extended)	*T4 bacteriophage*	3FOH	12.6	2.4	12	II, III, IV***	Aksyuk et al., 2009

* sheath protein model based on PDB entries 3LML and 3FOA fitted to cryo-EM reconstructions

** sheath assembly involves two proteins

*** domain I is present in the native protein but absent in the protease-treated sample used for crystallization

† VipA-N2/VipA-N3 contain N-terminal linker

2 Supplementary References

Aksyuk, A.A., Leiman, P.G., Kurochkina, L.P., Shneider, M.M., Kostyuchenko, V.A., Mesyanzhinov, V.V., and Rossmann, M.G. (2009). The tail sheath structure of bacteriophage T4: a molecular machine for infecting bacteria. EMBO J. *28*, 821–829.

Aksyuk, A.A., Kurochkina, L.P., Fokine, A., Forouhar, F., Mesyanzhinov, V.V., Tong, L., and Rossmann, M.G. (2011). Structural conservation of the Myoviridae phage tail sheath protein fold. Structure *19*, 1885–1894.

Arnaud, C.-A., Effantin, G., Vivès, C., Engilberge, S., Bacia, M., Boulanger, P., Girard, E., Schoehn, G., and Breyton, C. (2017). Bacteriophage T5 tail tube structure suggests a trigger mechanism for Siphoviridae DNA ejection. Nature Communications *8*, 1953.

Chang, Y.-W., Rettberg, L.A., Ortega, D.R., and Jensen, G.J. (2017). In vivo structures of an intact type VI secretion system revealed by electron cryotomography. EMBO Rep. *18*, 1090–1099.

Clemens, D.L., Ge, P., Lee, B.-Y., Horwitz, M.A., and Zhou, Z.H. (2015). Atomic structure of T6SS reveals interlaced array essential to function. Cell *160*, 940–951.

Fokine, A., Zhang, Z., Kanamaru, S., Bowman, V.D., Aksyuk, A.A., Arisaka, F., Rao, V.B., and Rossmann, M.G. (2013). The molecular architecture of the bacteriophage T4 neck. J. Mol. Biol. *425*, 1731–1744.

Ge, P., Scholl, D., Leiman, P.G., Yu, X., Miller, J.F., and Zhou, Z.H. (2015). Atomic structures of a bactericidal contractile nanotube in its pre- and postcontraction states. Nature Structural & Molecular Biology *22*, 377–382.

Kudryashev, M., Wang, R.Y.-R., Brackmann, M., Scherer, S., Maier, T., Baker, D., DiMaio, F., Stahlberg, H., Egelman, E.H., and Basler, M. (2015). Structure of the Type VI Secretion System Contractile Sheath. Cell *160*, 952–962.

Leiman, P.G., and Shneider, M.M. (2012). Contractile Tail Machines of Bacteriophages. In Viral Molecular Machines, (Springer, Boston, MA), pp. 93–114.

Lim, Y.T., Jobichen, C., Wong, J., Limmathurotsakul, D., Li, S., Chen, Y., Raida, M., Srinivasan, N., MacAry, P.A., Sivaraman, J., et al. (2015). Extended loop region of Hcp1 is critical for the assembly and function of type VI secretion system in Burkholderia pseudomallei. Sci Rep *5*, 8235.

Lin, Q.P., Gao, Z.Q., Geng, Z., Zhang, H., and Dong, Y.H. (2017). Crystal structure of the putative cytoplasmic protein STM0279 (Hcp2) from Salmonella typhimurium. Acta Crystallogr F Struct Biol Commun *73*, 463–468.

Mougous, J.D., Cuff, M.E., Raunser, S., Shen, A., Zhou, M., Gifford, C.A., Goodman, A.L., Joachimiak, G., Ordoñez, C.L., Lory, S., et al. (2006). A Virulence Locus of Pseudomonas aeruginosa Encodes a Protein Secretion Apparatus. Science *312*, 1526–1530.

Nováček, J., Šiborová, M., Benešík, M., Pantůček, R., Doškař, J., and Plevka, P. (2016). Structure and genome release of Twort-like Myoviridae phage with a double-layered baseplate. Proc. Natl. Acad. Sci. U.S.A. *113*, 9351–9356.

Pell, L.G., Kanelis, V., Donaldson, L.W., Howell, P.L., and Davidson, A.R. (2009). The phage lambda major tail protein structure reveals a common evolution for long-tailed phages and the type VI bacterial secretion system. Proc. Natl. Acad. Sci. U.S.A. *106*, 4160–4165.

Salih, O., He, S., Planamente, S., Stach, L., MacDonald, J.T., Manoli, E., Scheres, S.H.W., Filloux, A., and Freemont, P.S. (2018). Atomic Structure of Type VI Contractile Sheath from Pseudomonas aeruginosa. Structure *26*, 329-336.e3.

Wang, J., Brackmann, M., Castaño-Díez, D., Kudryashev, M., Goldie, K.N., Maier, T., Stahlberg, H., and Basler, M. (2017). Cryo-EM structure of the extended type VI secretion system sheath-tube complex. Nat Microbiol *2*, 1507–1512.

Zheng, W., Wang, F., Taylor, N.M.I., Guerrero-Ferreira, R.C., Leiman, P.G., and Egelman, E.H. (2017). Refined Cryo-EM Structure of the T4 Tail Tube: Exploring the Lowest Dose Limit. Structure *25*, 1436-1441.e2.
